# Anything New under the Sun? An Update on Modulation of Bioactive Compounds by Different Wavelengths in Agricultural Plants

**DOI:** 10.3390/plants10071485

**Published:** 2021-07-20

**Authors:** Marco Santin, Annamaria Ranieri, Antonella Castagna

**Affiliations:** 1 Department of Agriculture, Food and Environment, University of Pisa, I-56124 Pisa, Italy; marco.santin@agr.unipi.it (M.S.); anna.maria.ranieri@unipi.it (A.R.); 2 Interdepartmental Research Center “Nutraceuticals and Food for Health”, University of Pisa, Via del Borghetto 80, I-56124 Pisa, Italy

**Keywords:** photoreceptors, plants, bioactive compounds, ultraviolet, visible light, secondary metabolism

## Abstract

Plants continuously rely on light as an energy source and as the driver of many processes in their lifetimes. The ability to perceive different light radiations involves several photoreceptors, which in turn activate complex signalling cascades that ultimately lead to a rearrangement in plant metabolism as an adaptation strategy towards specific light conditions. This review, after a brief summary of the structure and mode of action of the different photoreceptors, introduces the main classes of secondary metabolites and specifically focuses on the influence played by the different wavelengths on the content of these compounds in agricultural plants, because of their recognised roles as nutraceuticals.

## 1. Introduction

Plants rely on an uncountable number of secondary metabolites during their lifespans in order to perform several fundamental functions, such as attracting pollinators, mechanical support, protection from solar UV radiation, deterrents against pests, pathogens, and herbivores, interaction with other plants, and response to environmental stimuli/stresses [1]. Thanks to a network of photoreceptors and the following complex signalling routes, the different light wavelengths may impact the content of these metabolites by up- or downregulating specific sets of biosynthetic and regulatory genes.

## 2. Photoreceptors

Light and plants are an inseparable pair. Light—in addition to playing a crucial role as the energy source for photosynthesis—controls a wide variety of processes during the whole plant life cycle, from seed germination to senescence. Therefore, plants have developed fine-tuning strategies to differentially perceive the wavelengths of the solar spectrum that reach the Earth’s surface, and to detect light intensity and direction. The mechanisms that allow plants to respond and adapt to the changing light environment involve several photoreceptors that perceive wavelengths from ultraviolet (UV) to far-red, and transfer the information through a downstream complex network of signals.

Generally, photoreceptors control gene expression by regulating the activity and stability of transcription factors [2,3,4,5] that culminate with modifications at transcriptional, translational, post-translational, and enzymatic levels. Such molecular and biochemical responses determine a rearrangement in the plant’s primary and secondary metabolism as an adaptation strategy towards specific light conditions.

**Figure 1:**
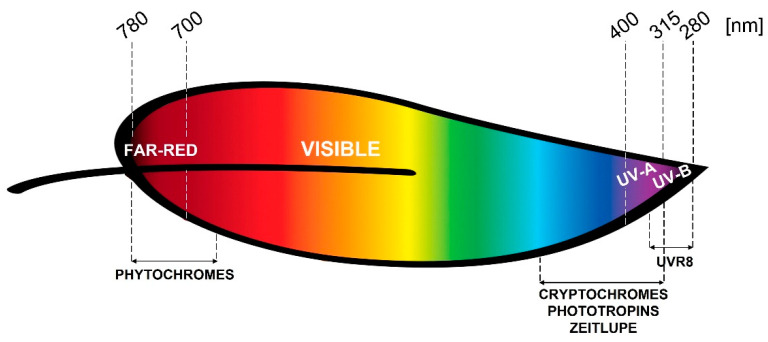
Wavelength ranges within the solar spectrum perceived by the different photoreceptors in plants.

As depicted in Figure 1, phytochromes (phy) are responsible for red/far-red perception, and cryptochromes (cry), phototropins (phot), and zeitlupe are the receptors of blue light. Cryptochromes also perceive green wavelengths. The UVR8 receptor is specifically involved in UV-B sensing, while UV-A is perceived by the blue light receptors, though very recently the involvement of UVR8 in mediating UV-A perception was demonstrated in Arabidopsis [6]. Despite plant responsiveness to green light being observed, presently, there is less known about the mechanism at the basis of green or yellow light perception.

Although the implication of phototropins, cryptochromes, and phytochromes in absorbing certain light ranges has been recognised for decades, the development of new technologies, in terms of molecular and cell biology, photobiology, and biochemistry, together with an integrated approach that combines different research fields, e.g., biology and ecology, have uncovered structures, functions, implications, and metabolic networks of photoreceptors that were still unknown.

The following section will briefly summarise the mechanisms of light perception by the different photoreceptors.

### 2.1. Phytochromes

Phytochromes are dimeric kinase proteins involved in several plant processes, such as seed germination, de-etiolation, stomata development, stem growth, pigmentation, flowering, senescence, and shade avoidance [7,8]. Phytochromes are the specific red/far-red photoreceptors, though they are also known to absorb blue light and to regulate blue light response [9]. The hydrophilic apoproteins, synthesised in the cytoplasm, links a tetrapyrrole chromophore (phytochromobilin), giving rise to the biologically inactive (Pr) form [10,11]. After red light absorption (around 665 nm), Pr is converted to the active (Pfr) form and translocates into the nucleus [12]. Following far-red irradiation (around 730 nm), Pfr is quickly converted back to the Pr form. Dark conditions also slowly revert Pfr to Pr form.

The apoprotein contains an N-terminal photosensory module (PSM), in turn, comprising an N-terminal extension (NTE) and the three domains: period/Arnt/SIM (PAS), cGMP phosphodiesterase/adenylyl cyclase/FhlA (GAF), and phytochrome-specific domain (PHY). A conserved cysteine residue of the GAF domain covalently binds the chromophore, which also interacts non-covalently with NTE, PHY, and PAS domains. The C-terminal module (CTM) consist of two PAS domains and a histidine kinase-related domain (HKRD) [13]. Upon red light perception, a Z to E isomerization of the chromophore occurs, triggering the structural modifications of the protein that allow its transport into the nucleus and the subsequent interaction with transcription factors of the PHYTOCHROME INTERACTING FACTOR (PIF) family and ubiquitin E3 ligase complexes. Specifically, Pfr-dependent phosphorylation of PIFs triggers their degradation via the ubiquitin 26S proteasome pathway; thus, removing the negative regulators of photomorphogenesis. Pfr also induces the disassembly of the ubiquitin E3 ligase complex, resulting in the accumulation of transcription factors, such as HY5 (ELONGATED HYPOCOTYL 5), a promoter of photomorphogenic development. Pfr also possesses an autocatalytic activity leading to the phosphorylation of the phytochrome itself [14,15]. This mechanism provides an instrument for attenuation of the phyA-mediated light signalling by accelerating phy protein degradation, while phyB phosphorylation leads to a reduced signalling via accelerated dark reversion [16].

### 2.2. Cryptochromes

Cryptochromes are blue/UV-A photoreceptors involved in many physiological responses, such as photomorphogenesis, seedling development and de-etiolation, flowering, circadian rhythms [17,18], plant stress responses to pathogens, and shade avoidance [19]. Some responses to green wavebands are cryptochrome-dependent as well [20]. Cryptochromes exist as inactive monomers in dark conditions. The apoprotein can bind to two different chromophores: a flavin (in the form of flavin adenine dinucleotide, FAD) absorbing at 450 nm, and a pterin (5,10-methenyltetrahydrofolic acid, MTHF) absorbing at 380 nm [21]. Upon light absorption, conformational changes occur, leading to homo-oligomerization, a modification that changes the affinity with signalling proteins to form various cryptochrome complexes, generically referred to as the cryptochrome complexome [22]. Cry1 and Cry2 are both located in the nucleus, and Cry3 probably acts in chloroplasts and mitochondria [5]. While the photoexcited Cry1 migrates in the cytosol, and is stable and functions under high-fluence irradiance, Cry2 remains in the nucleus [23], is quickly downregulated by blue light, and works at lower light intensities [24]. A detailed description of the different mechanisms of action of cryptochromes and the signalling components involved in the Cry signal transduction pathway is reported in Mishra and Khurana [21].

### 2.3. Phototropins

Phototropins—differently from the other photoreceptors—are primarily located on the plasma membrane. Phototropins have a serine/threonine kinase domain and two chromophore-binding N-terminus LOV (light, oxygen, and voltage) domains [25]. Following light absorption by the flavin mononucleotide (FMN) chromophores bound to LOV1 and LOV2, a conformational change occurs. This activates the kinase domain and phototropin undergoes autophosphorylation [26], a mandatory mechanism for the phototropin function [26,27].

Phototropins mediate plant responses to blue light and UV-A radiation at the subcellular, cellular, organ, and tissue level, controlling primarily those processes involved in promotion of photosynthetic light absorption and utilization, such as phototropism, stomata opening, chloroplast movement and orientation, and leaf expansion and flattening [26].

### 2.4. Zeitlupe Family

The zeitlupe photoreceptors are localised in the cytosol or in the nucleus and perceive UV-A and blue radiations. The three members of this family, namely zeitlupe (ztl), flavin-binding kelch repeat F-box 1 (fkf1), and LOV kelch protein 2 (lkp2), present a LOV domain at the N-terminus, an F-box, and six kelch repeats at their C-terminus [18]. Members of the zeitlupe family participate in the photoperiodic control of flowering, in the regulation of the circadian clock and the control of hypocotyl elongation [28]. These actions are carried out thanks to the participation of the F-box domain in the E3 ubiquitin ligase Skp–Cullin–F-box (SCF) complex, which triggers a controlled light-mediated protein degradation [18].

### 2.5. UVR8

The UV RESISTANCE LOCUS 8 (UVR8) is the specific receptor of UV-B radiation [29], though very recently, a role in the perception of short wavelength UV-A (315–350 nm) was demonstrated in Arabidopsis [6]. UVR8, in its inactive form, is a dimeric protein located in the cytosol. Upon light perception, UVR8 monomerizes and migrates into the nucleus, where it initiates the signal transduction, ultimately leading to up- or downregulation of target genes. This photoreceptor does not bind a chromophore. Light sensing is carried out by the UVR8 tryptophan residues. In particular, the tryptophan residue in the position 285 (Trp-285) seems to be the key element in UVR8 monomerization. Once it enters the nucleus, UVR8 monomer interacts with CONSTITUTIVELY PHOTOMORPHOGENIC 1 (COP1) [30], dissociating the COP1-SPA core from the CUL4-DDB1-based E3 ubiquitin ligase complex. Therefore, HY5 accumulates and promotes the transcription of many UV-B-induced genes [31,32] involved in the photomorphogenic responses and UV-B acclimation [33,34,35]. Interestingly, among the UVR8-induced gene, there are the REPRESSOR OF UV-B PHOTOMORPHOGENESIS (RUP) 1 and 2. RUP1 and RUP2 proteins promote UVR8 dimerization, thus acting as negative regulators of UV-B signalling [36].

## 3. Signal Transduction Pathways

Independently from the light quality and kind of photoreceptor involved in light perception, the downstream event proceeds via a complex network of early signalling factors, central integrators, and final effectors. Please refer to some recent reviews [37,38,39,40] for a detailed summary of the current knowledge of the transcriptional network and mechanisms regulating the response to the different light spectral composition. Interestingly, CONSTITUTIVE PHOTOMORPHOGENIC 1 (COP1), which promotes the proteasome-mediates degradation of key factors involved in light signalling, is involved in the response to any light radiation, from UV to far-red wavelengths [41]. Similarly, the transcription factor ELONGATED HYOCOTYL 5 (HY5) has a central role as a final effector of all the light-dependent signalling routes, being able to bind to the promoters of about 4000 genes in Arabidopsis [41].

**Figure 2:**
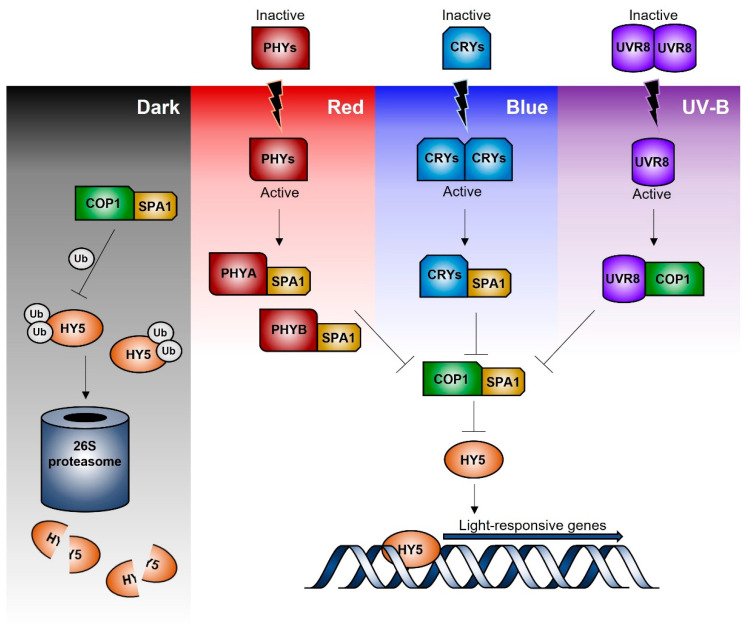
Simplified plant perception mechanisms of different types of solar radiation (dark, red, blue, and UV-B), together with the intracellular rearrangements leading to the transcription of specific light-responsive genes.

Figure 2 represents a simplified scheme of the signal transduction pathways leading to gene regulation in response to blue, red/far-red, and UV-B radiation. Briefly, under dark conditions, COP1/SPA (suppressor of Phytochrome A) ubiquitin ligase complex promotes the ubiquitination and degradation of HY5 via the 26S-proteasome pathway [39]. Upon light perception, the active blue- and red/far-red-photoreceptors (cryptochromes and phytochromes) interact with the COP1/SPA complex binding to SPA; thus, leading to COP1 disassembly and migration outside the nucleus. This prevents HY5 ubiquitination and subsequent degradation, so that HY5 may bind the promoter sequence of the light inducible target genes. Similarly, UVR8, after UVB-induced monomerization, can bind to COP1, leading to a functional disruption of the COP1/SPA complex and a consequent HY5 stabilization and functioning [33,42].

## 4. Plant Metabolism and Light

This review specifically focuses on the influence of the different light radiations, from red–far-red to UV-B, on the main classes of secondary metabolites, such as phenolic compounds, terpenoids, tocopherols, glucosinolates, and ascorbic acid in agricultural plant species, because of the recognised role that these compounds generally play as promoters of human wellness [43,44,45,46]. UV-C radiation was reported to modulate accumulation of health-promoting compounds in different plants and fruits of food interest, such as tomato fruit [47], bean seedlings [48] and peanut sprouts [49], this review exclusively discusses the effects of those wavelengths that reach the Earth’s surface, and to which plants have adapted fine-tuning perception mechanisms and consequent molecular and biochemical responses through evolution.

**Figure 3:**
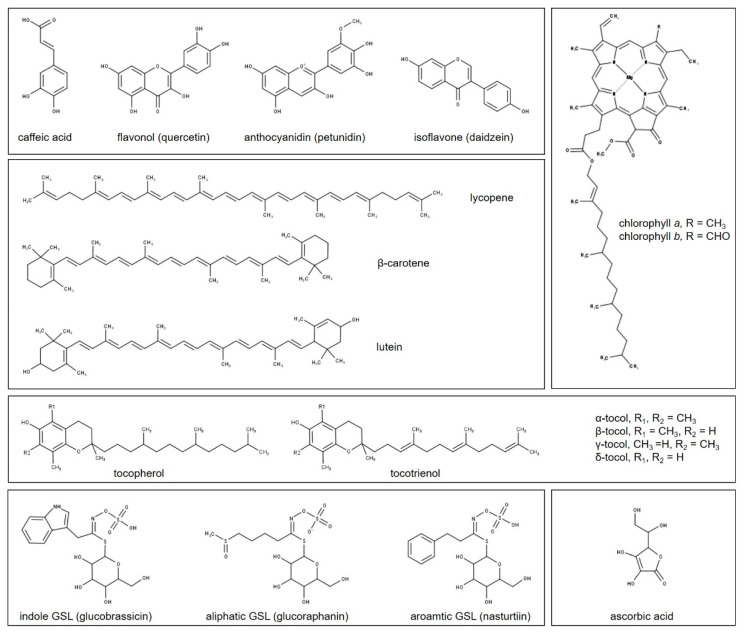
Examples of secondary metabolites whose content is modulated by the different light wavelengths: phenolic compounds (hydroxycinnamic acids and flavonoids), carotenoids, chlorophylls, tocopherols and tocotrienols, ascorbic acid, and glucosinolates. Marvin was used for drawing the chemical structures (Marvin 17.21.0, ChemAxon. Budapest, Hungary).

Examples of the chemical structures of the biomolecule classes described below, and whose content is under the control of the different light wavelengths, are presented in Figure 3.

### 4.1. Phenolic Compounds

Phenolic compounds constitute an extremely huge family (more than 8000 members currently found) of secondary metabolites, which is ubiquitous in vascular plants and bryophytes [50]. Structurally, phenolic compounds consist of one (phenols) or more (polyphenols) aromatic rings linked with one or more hydroxyl groups, and possible other functional substituents (e.g., glycosides), whose number and position within the molecule determine their specific activity. According to their carbon skeleton, the wide class of phenolics can be classified as: C6 (simple phenol, benzoquinones), C6-C1 (phenolic acid), C6-C2 (acetophenone, phenylacetic acid), C6-C3 (hydroxycinnamic acids, coumarins, phenylpropanes, chromones), C6-C4 (naphthoquinones), C6-C1-C6 (xanthones), C6-C2-C6 (stilbenes, anthraquinones), C6-C3-C6 (flavonoids, isoflavonoids), (C6-C3)2 (lignans, neolignans), (C6-C3-C6)2 (biflavonoids), (C6-C3)n (lignins), (C6)n (catechol melanins), (C6-C3-C6)n (condensed tannins) [51]. All the plant phenolics originate from pentose phosphate, shikimate, and phenylpropanoid pathways [52].

In recent decades, phenolics have gained popularity with consumers thanks to their benefits for human health [53], particularly due to their anti-allergenic, anti-cancerogenic, anti-atherogenic, anti-inflammatory, anti-microbial, antioxidant, anti-thrombotic, cardioprotective, and vasodilatory properties [54,55,56,57]. The main sources of phenolics in the human diet are fruit and vegetable products, although they have also been found in teas, wine, chocolate, herbs and spices, grain, and seeds [58,59,60,61,62] Among phenolics, the widest subfamily is represented by flavonoids, which counts more than 7000 different molecules, and their number keeps on increasing [63]. Flavonoids are the phenolics exhibiting the strongest pharmacological activity and antioxidant capacity [64,65] and, therefore, their consumption within the diet is strongly recommended. Flavonoid subclasses, which differ based on the type of substituents on the central ring of the molecule, are flavonols, flavones, flavanones, flavan-3-ols, isoflavones, and anthocyanidins, while the substituents linked to the aromatic rings (e.g., hydroxyl groups or post-translational modifications such as glycosylation, sulphonation, and acylation) determined the individual members of each flavonoid subclass.

### 4.2. Terpenoids

Terpenoids constitute the largest family of secondary metabolites, counting more than 60,000 members [66]. Although they show an extreme variability in chemical structures, all terpenoids derive from the same five carbon isoprene (C5) units, whose number is the main criteria for their classification: C5 (hemiterpenoids), C10 (monoterpenoids), C15 (sesquiterpenoids), C20 (diterpenoids), C25 (sesterterpenoids), C30 (triterpenoids), C40 (tetraterpenoids), and C > 40 (polyterpenoids). Terpenoids C5 precursors, the isopentenyl diphosphate (IPP). and its isomer dimethylallyl diphosphate (DMAPP), are synthesised through two distinct and independent pathways, the mevalonic acid (MVA), and the methylerythritol phosphate (MEP) pathways, which took place in the cytosol and in the plastids, respectively [66,67,68,69].

According to their chemical structures, terpenoids fulfil essential functions during plant life as, e.g., direct/indirect defensive compounds against biotic stressors, deterrent towards herbivores, photosynthetic pigments, signalling molecules mediating plant-plant, and plant–environment interaction [66,69,70,71].

As health-promoting compounds in humans, terpenoids have been discovered to have strong antifungal, antimicrobial, antiviral, anti-inflammatory, immunomodulatory, gastroprotective, and anticarcinogenic properties [66,72,73,74]; thus, their use for medicinal and pharmaceutical purposes has increased in the last decades. Besides, most of them, being volatile, contribute toward giving the peculiar flavour and aroma of many fruits and vegetables and their food derivatives, thus influencing the overall organoleptic quality and marketability of plant-based products [72,75,76,77,78,79].

Among terpenoids, carotenoids represent a large group of metabolites (more than 600) playing a key role both in plant organisms and for human health [80]. Carotenoids are natural pigments ranging from yellow to red colours, which have been found in animals, plants, and microorganisms [81,82]. Furthermore, in photosynthetic organisms, their role in the pigmentation is crucial for photoprotection and light absorption mechanisms, thus contributing to the overall photosynthetic process [83]. Humans cannot synthesize carotenoid compounds; therefore, they must be introduced through the diet or via supplementation [84]. Few carotenoids (particularly β-carotene, lutein, and lycopene) were reported to have concrete benefits for human health, being associated with a reduced risk of several pathologies, such as cardiovascular diseases, different types of cancer, immunodeficiencies, fertility, and eye-related problems [85,86,87,88,89,90].

### 4.3. Tocopherols and Tocotrienols

Vitamin E represents another essential bioactive compound with beneficial effects in human metabolism. Chemically, vitamin E refers to four tocopherols (α-,β-,γ-, and δ-) and four tocotrienols (α-,β-,γ-, and δ-), which are altogether known as tocochromanols or tocols, and are all characterised by a chromanol headgroup and a prenyl side chain [91]. The tocotrienols, unlike the tocopherols, exhibit three unsaturations in the hydrophobic chain, while differences among tocopherols and tocotrienols are due to the number and position of alkyl substituents on the chromanol moiety [92]. Vitamin E compounds exhibit a hydrophobic nature; thus, they are present in lipidic structure within the cells (e.g., cell membranes), fat deposits, poly- and mono-unsaturated fatty acids (PUFA and MUFA, respectively), and lipoproteins [92].

Vitamin E has been widely studied due to its high antioxidant activity, especially preventing the oxidation of mono- and poly-unsaturated lipids. In addition, vitamin E compounds were shown to have hypolipidemic, antiatherogenic, antihypertensive, neuroprotective, anti-inflammatory, and many other beneficial effects for human health [93,94,95,96,97]. The main plant sources of tocopherols and tocotrienols are seeds (especially oilseeds) and nuts. In addition, they can be found in many plants and fruits, although their concentrations are limited due to their low lipid content [98].

### 4.4. Ascorbic Acid

Another essential antioxidant micronutrient, not only for humans, but also for other animals and plant organisms, is the ascorbic acid, also known as vitamin C. All plants and many animals, including several mammals, have the ability to synthesize the vitamin C molecule, although others, including primates, guinea pigs, humans, and different bird species, have lost such capacity through evolution [99]. Ascorbate molecule is a C6 compound that derives through different biosynthetic pathways, such as the D-glucose, L-galactose, uronic acid, L-gulose, and myo-inositol pathways [100], but the vitamin C biosynthesis relies mainly on the Smirnoff–Wheeler (SW) pathway [101]. Moreover, ascorbate availability within the cell also depends on its recycling process, which is ensured by the Foyer–Halliwell–Asada cycle [102,103].

Vitamin C, like the majority of the hydrosoluble vitamins, participates as a cofactor for many enzymes, e.g., members of the mono- and dioxygenases family [99], essentially contributing to the maintenance of the cell redox state, together with several other antioxidant molecules and enzymes. In plants, vitamin C is involved in many pathways and processes, e.g., the xanthophyll cycle, the flavonoids, and the glucosinolates pathways, and in the biosynthesis of plant hormones, such as ethylene, gibberellins, and abscisic acid [104,105,106,107,108]. Studies on the role of vitamin C role and its benefits in humans started when it was first noticed that vitamin C deficiency determined a potentially lethal disease called scurvy [109], negatively affecting the immune system, the collagenous architecture, and the regeneration process from wounds. Moreover, pharmacological effects of ascorbic acid against cancer and cardiovascular diseases were also observed [110,111]. The main dietary sources of vitamin C are fresh fruits and vegetables; therefore, their consumption has been widely encouraged by the main food and health organisations (e.g., the Food and Nutrition Board of the National Academy of Sciences, the European Food Safety Authority (EFSA), and the Food and Drug Administration (FDA)) throughout the years, and vitamin C deficiency symptoms have progressively reduced worldwide.

### 4.5. Glucosinolates

Glucosinolates (GSLs) are an important class of secondary metabolites, widely spread within all of the species of the order Brassicales, including the model plant *Arabidopsis thaliana*. The backbone of a GSL molecule consists of a sulphonated oxime group bound to a thioglucose substituent, and an amino acid-derived R group. GSLs include more than 200 compounds, classified as aromatic (from tyrosine or phenylalanine), aliphatic (from methionine, valine, alanine, leucine or isoleucine), or indolic (from tryptophan), according to the R group of the molecule [112]. Their concentration within the Brassica species can vary, extremely, based on several internal (e.g., genotype and developmental stage of the plant) and external (e.g., temperature, fertigation, light, cultivation method, storage condition) factors [113,114,115,116,117,118].

GSLs represent essential defensive molecules against biotic factors, due to their strong antibacterial and antifungal properties [119,120]. However, to be converted into their active, toxic form (called isothiocyanates), thus performing their biological function, the thioglucose residue of the GSL molecule needs to be removed by the activity of specific β-thioglucosidases called myrosinases [119]. Due to the high toxicity of the aglycones, myrosinases and GSLs are located in distinct intracellular compartments to avoid accidental formation of isothiocyanates. This way, the plant ensures that the formation of such defensive molecules occurs only when a plant tissue is damaged, e.g., by the attack of pests and herbivores. Moreover, their role against several abiotic stresses, such as drought and salinity, has been elucidated [121,122,123,124]. In humans, many studies have demonstrated the beneficial effects of GSLs as antibacterial, antifungal, antitumoral, and antioxidant compounds [125,126,127].

## 5. Red and Far-Red Light

Although the impact of red/far-red light application on plants is well known in terms of growth performance, morphological and physiological parameters, and productivity, its influence on plant secondary metabolites (e.g., phenolics and terpenoids) are controversial. Examples of the variegated effects induced by these radiations are reported below and summarised in Table 1.

### 5.1. Phenolics

Increased content of total phenolics and flavonoids was observed in common buckwheat (*Fagopyrum esculentum* Möench) sprouts grown under red light LEDs (625 nm, 16 h a day, 7 days) as compared to dark-grown sprouts [128]. However, their content was lower than under fluorescent or blue light, the latter inducing the highest accumulation of these molecules.

A study conducted on two varieties of lettuce (*Lactuca sativa* L.), a red leafy one (cv. Sunmang) and a green leafy one (cv. Grand Rapid TBR) grown under red light (655 nm, 171 ± 7 μmol m^–2^ s^–1^, light-emitting diode (LED) lighting sources), for 4 weeks, showed a significant decrease in total phenolics and antioxidant capacity in the red leaf cultivar, while total flavonoids were significantly higher compared to the control level [129]. On the contrary, the green leafy variety did not show any variation in terms of total phenolics, flavonoids, and antioxidant capacity, indicating a genotype-dependent responsiveness to red light of this biosynthetic pathway. Another study on the same green leafy variety of lettuce confirmed the inefficacy of a 25-day red-light treatment (661 nm, 200 μmol m^−2^ s^−1^, LED lighting sources) in modifying the content of total phenolics and flavonoids [130]. Effectiveness of red-light irradiation in improving the nutraceutical quality of red leafy lettuce was also previously found by Li and Kubota (2009) [131], on the cultivar Red Cross. The authors observed a significantly higher content of phenolics in plants grown for 12 days under cool white fluorescent lamps supplemented with red light (658 nm, 130 ± 10 µmol m^−2^ s^−1^, LED lighting sources). Despite such an increase, anthocyanins concentration was unchanged in respect to the control. Moreover, when the same lettuce cultivar was irradiated with supplemental far-red light (734 nm, 160 ± 5 µmol m^−2^ s^−1^, LED lighting sources), anthocyanin concentration decreased, although the phenolics level did not vary.

**Table 1:** Biochemical responses of crops and plants of food interest to red and far-red light wavelengths considered in this review. Tot, total phenolics; Flav, flavonoids; Ant, anthocyanins; AC, antioxidant capacity; T, terpenoids; AA, ascorbic acid; TP, tocopherols; GSL, glucosinolates. For each plant species and cultivar, and for each secondary metabolite or metabolic class considered, the symbols “↓”, “↑” and “=” mean a decrease, increase or no variations, respectively, compared to the control plants of each study.

Species	Cultivar	Phenolics			AC	T	AA	TP	GSL	Ref.
		Tot	Flav	Ant						
Red leafy lettuce (*Lactuca sativa.* L.)	Sunmang	↓	↑		=					[129]
	Red Cross	↑/=		=/↓		=/↓	=			[131]
	Red Fire					↓/=				[132]
Green leafy lettuce (*Lactuca sativa.* L.)	Grand Rapid TBR	=	=		=					[129,130]
	Thumper					↓		↑		[133]
Lamb’s lettuce (*Valerianella locusta* L.)	Noordhollandse			↑						[134]
	Holländisher			↓						[119]
Pea (*Pisum sativum* L.)					↑	=/↑				[135]
	Meteor	↑	↑		=		↑			[136]
Amaranth (*Amaranthus cruentus* L.)	Red Army	↓	↑		=		↑			[136,137,138]
Basil (*Ocimum basilicum* L.)	Genovese	↑	=/↑		↑	↓	↓			
Kale (*Brassica oleracea* L.)	Red Russian	↑	↑		↑		↑		↑	[136,139]
Chinese kale (*Brassica oleracea* L. var. *alboglabra* Bailey)	DSCH						↑			[140]
	DFZC								=	[141]
Broccoli (*Brassica oleracea* L.)		↑	↑		=		↑			[136,142]
Mustard (*Brassica juncea* L.)	Red Lion	↑	↓		↑		↑			
Tatsoi (*Brassica rapa* L.)	Rosularis	↑	↑		↑		=			
Orach (*Atriplex hortensis* L.)		↑			=					
Borage (*Borago officinalis* L.)		↑	↓		↑		↓			
Beet (*Beta vulgaris* L.)	Bulls Blood	↑	↓		↓		=			
Parsley (*Petroselinum crispum* Mill.)		↑	↑		↑		=			
Parsley (*Petroselinum crispum* Mill.)						↑				[137]
Strawberry (*Fragaria × Ananassa*)	Elsinore	=	↓		=					[138]
Cranberry (*Vaccinium macrocarpon* Ait)	Early Black			↑						[143]
Red clover (*Trifolium pratense* L.)						↓/=				[144]
Buckwheat (*Fagopyrum esculentum*)	Möench	↑	↑							[128]
Spinach (*Spinacia oleracea* L.)	Okame					↓				[132]
Wheat (*Triticum aestivum* L.)		=								[145]
Tartary buckwheat (*Fagopyrum tataricum* Gaertn.)	Hokkai T8					↓				[146]
Cowpea (*Vigna unguiculata* L. Walp.)						↓				[147]
Bilberry (*Vaccinium myrtillus* L.)				↑						[148]
Tomato (*Solanum lycopersicum* L.)	Red Ruby					↑				[47]
Tea leaves (*Camellia sinensis*)	Jinxuan					↑				[149]
Pak choi (*Brassica rapa* ssp. chinensis)						↓				[150]
(*Brassica rapa* ssp. pekinensis)	Chiifu								↑	[151]
Satsuma mandarin fruit (*Citrus unshiu* Marc.)						↓/↑				[152]

A study conducted on cranberry plants grown under either red (photon fluence rate of 12 μmol m^−2^ s^−1^, fluorescent lamps as lighting sources filtered through a red plastic sheet) or far-red (photon fluence rate of 5 μmol m^−2^ s^−1^, halogen double ended quartz bulbs as lighting sources filtered through a 3 mm far-red plastic) light showed that fruit anthocyanins were significantly higher when compared to fruits grown under white light (by 6.44 and 3.68-fold, respectively, for red and far-red light) [143].

Similarly, when bilberries (*Vaccinium myrtillus* L.), plants were exposed to monochromatic red light (7.8 μmol m^−2^ s^−1^) during the berry ripening period, a significant increment of total anthocyanins occurred, due to the positive effect of this radiation on petudinins and delphinidins, while peonidins decreased, and cyanidins and malvidins were unaffected [148]. This finding underlines an interesting aspect of the light–phenolic interaction, i.e., the diversity of response to the same stimulus shown by different subclasses of molecules belonging to the same metabolic class. A similar phytochemical specificity of response was also observed in wheat (*Triticum aestivum* L.) sprouts grown under a 16-h light/8-h dark photoperiod under white, red, or blue light, for up to 12 days. Specifically, red light, at the end of the growing period, did not lead to a significant increase in the content of total phenylpropanoids in comparison to white light, but modified their composition, inducing an increase in quercetin and a decrease in 4-hydroxybenzoic acid [145].

Phenolic compounds, and flavonoids in particular, are recognised for their beneficial influence on human health, thanks to their ability to reduce the radical accumulation via the radical-scavenging or chain-breaking activities; thus, preventing the oxidation of many biomolecules [153].

A study by Wu et al. [135] found that antioxidant activity of pea (*Pisum sativum* L.) seedlings grown under red light (625–630 nm, 128 ± 4.38 lx, LED lighting sources) increased when compared to the seedlings grown under white light. Diversity of responses considering antioxidant properties was clearly highlighted by the study of Samuolienė et al. (2012) [136], which compared the impact of a supplementary short-term red LEDs lighting (638 nm, 170 µmol m^−2^ s^−1^) on microgreen from different plant species (amaranth (*Amaranthus cruentus* L.), basil (*Ocimum basilicum* L.), tatsoi (*Brassica rapa* L.), mustard (*Brassica juncea* L.), spinach (*Spinacia oleracea* L.), broccoli, kale (*Brassica oleracea* L.), borage (*Borago officinalis* L.), beet (*Beta vulgaris* L.), parsley (*Petroselinum crispum* Mill.), and pea). Enhanced antiradical activity was observed in seven of the ten species tested, and the treatment determined an increase in total phenolics in almost all the plant species, ranging from 9.1% in mustard to 40.8% in tatsoi seedlings. Amaranth was the only species registering a decrease in phenolic concentration (−14.8%). However, the effect of red light on total anthocyanins was more variable, displaying an overall increase in broccoli, kale, amaranth, tatsoi, parsley, and pea (from 14.6% in pea to 45.1% in broccoli), while in borage, mustard and beet total anthocyanins underwent a marked decrease (from 43.3% in beet to 51.8% in borage).

Positive effect of red-light irradiation (635–700 nm, 200 µmol m^−2^ s^−1^, LED lighting sources) was observed also on basil leaves, in which the antioxidant capacity, the total phenolics, and the flavonoid concentration increased by 14, 30, and 52%, respectively [138]. In the same study, however, it was reported that the red-light treatment did not affect phenolics content and antioxidant capacity of strawberry (*Fragaria* × *ananassa*) fruit, even leading to a decreased flavonoid content. The few examples reported above witness the potential of red and far-red to interact with the phenylpropanoid biosynthesis, but, at the same time, highlight the complexity of this response, that involves a highly co-ordinated control of regulatory and structural biosynthetic genes in a species- and tissue-specific way.

### 5.2. Terpenoids and Chlorophylls

As for phenolics, the effect of red/far-red light on terpenoids, in particular on carotenoids, is highly variable, and strictly depends on the plant species and cultivar considered.

A 7-day exposure of red clover (*Trifolium pratense* L.) sprouts to red-light (630 nm, 150 μmol m^−2^ s^−1^, LEDs as lighting sources) induced a significant decrease in zeaxanthin concentration, while β-carotene and lutein were unaffected by the treatment [144]. A negative impact of this radiation on β-carotene concentration (−42.5%) was instead observed in Romaine green baby leaf lettuce (cv. Thumper) treated with supplemental red light (638 nm, 150 μmol m^−2^ s^−1^, LEDs lighting sources) for 3 days [133]. These results differed from the ones by Li and Kubota (2009) [131], who found that 12 days of supplemental far-red light, but not red-light, irradiation determined a decrease in xanthophylls and β-carotene concentration in “Red Cross” baby leaf lettuce. Moreover, red light (380 μmol m^−2^ s^−1^, LED lighting sources) was ineffective in modifying the carotenoid content of another lettuce cultivar (“Red Fire”) when compared to white light [132].

A dramatic decrease of all carotenoids (13Z-β-carotene, E-β-carotene, 9Z-β-carotene, α-carotene, and lutein), with the exception of zeaxanthin, was observed in cowpea (*Vigna unguiculata* L. Walp.) sprouts exposed to red light (660 nm, 50 μmol m^−2^ s^−1^) irradiation for 2 weeks [147]. Similarly, tartary buckwheat (*Fagopyrum tataricum* Gaertn., cv. Hokkai T8) sprouts exposed to red light (660 nm, 50 μmol m^−2^ s^−1^) for 16 h a day accumulated lower amounts of carotenoids as compared to sprouts grown under white light, again with the exception of zeaxanthin [146]. However, when considering etiolated pea seedlings, a 96 h-red-light irradiation induced a significant increase in β-carotene content, though only in leaf tissues, but not in stems [135]. An increased β-carotene content was also reported by Samuolienė et al. [137] in parsley (*Petroselinum crispum*) microgreens under additional (638 nm) or unique (638 or 665 nm) red lightning. However, the same conditions led to decreased β-carotene content in basil (cv Sweet Genovese) microgreens and lowered the lutein accumulation on both species. It is therefore evident that, with few exceptions, red light never plays a positive effect on carotenoid accumulation in leafy vegetables and sprouts. This aspect should be taken in consideration anytime the interest of the production chain is the obtainment of high-quality plant foods. Carotenoids contribute in fact to the aesthetical aspect of the product, which is the first attribute that orientates the consumer’s choice. Of pivotal importance are also the nutraceutical properties of carotenoids, due to their general antioxidant power and the provitamin A activity of α- and β-carotene, and β-cryptoxanthin [81,154,155].

Irradiation on fruits with red wavelengths was more successful as compared to leafy vegetables. Specifically, a significant increment of β-cryptoxanthin level, though still accompanied by a decreased content of β- and α-carotene, was evident in Satsuma mandarin (*Citrus unshiu* Marc.) fruits treated for 6 days with 50 μmol m^−2^ s^−1^ of red (660 nm) light [152], and a noteworthy increase of lycopene, but not of β-carotene, concentration was induced in tomato (*Solanum lycopersicum* L. cv. Red Ruby) fruits after 21 days of post-harvest exposure to red light (610–750 nm) for 24 min per day [47].

Interestingly, a 3-day exposure of tea leaves (*Camellia sinensis* var. Jinxuan) to red light (660 nm, 70–80 μmol m^−2^ s^−1^) increased the volatile terpenes (such as geraniol, linalool, linalool oxide, and diendiol I) levels in respect to the dark conditions [149], suggesting that monochromatic irradiation in pre-harvest could represent a powerful and promising strategy to modify the aroma of tea leaves. However, a prolonged exposure (14 days) did not affect or even decreased these volatiles and a post-harvest irradiation with the same light irradiance was less efficient than in pre-harvest, probably due to the limited irradiation period (4 h at maximum).

Chlorophylls, besides giving the typical green pigmentation to leafy vegetables, may contribute to the antioxidant potential of the produce [156]. Unfortunately, red light seems to play a negative effect on chlorophyll concentration, as indicated by the study carried out by Son and Oh (2013) [129], who reported that red-light-irradiated lettuce exhibited a significantly lower chlorophyll content in both the red leafy cv, “Sunmang”, and in the green leafy one, “Grand Rapid TBR”. Similarly, Ohashi-Kaneko et al. [132], observed a significant reduction of chlorophyll concentration in lettuce (cv Red Fire) and Komatsuna (*Brassica campestris* L. cv. Komatsuna) irradiated with red light (380 μmol m^−2^ s^−1^, LED lighting sources). The same treatment, however, did not have any effect on spinach (*Spinacia oleracea* L. cv. Okame) [132]. However, Li and Kubota (2009) [131] found that 12 days of supplemental far-red light, but not red-light, irradiation determined a decrease in chlorophyll concentration in “Red Cross” baby leaf lettuce. Recently, a decrease in chlorophyll concentration was observed also in pak choi (*Brassica rapa* ssp. chinensis) sprouts cultivated under red (peak at 663 nm) LEDs [150].

### 5.3. Other Secondary Metabolites

The influence of light spectral quality on plant secondary metabolism is not limited to the biosynthesis of phenolic and terpenoids compounds, but it may also affect other bioactive compounds, as tocopherols, ascorbic acid, glucosinolates, etc. The potential modification of different classes of metabolites by changing the light environment is particularly important because, despite the attention is often focused on specific compounds, the nutraceutical power of a plant food derives from its unique combination of many different hydrophilic and lipophilic molecules.

Tocopherols are reported to be influenced by red light. Exposure to supplemental red light (638 nm, 150 μmol m^−2^ s^−1^, LEDs lighting sources, 3 days) was effective in significantly increasing α- and γ-tocopherols of Romaine green baby leaf lettuce (cv. Thumper) [133]. The same authors [137] also detected significant accumulation of α-tocopherol in basil microgreens grown under increased or sole red radiation (638 nm). However, increased red radiation lowered the α-tocopherol content of parsley microgreens, which was instead incremented when cultivation occurred with sole red lightning. It is therefore evident that, as observed for phenolic compounds, tocopherols are also influenced by red radiation in a species-depending way.

Variability of response was evident also for ascorbic acid. No effect on its concentration was indeed detected in “Red Cross” baby leaf lettuce treated with either red or far-red irradiation [131]. Similarly, a 3-day exposure to supplemental red-light (638 nm, 170 µmol m^−2^ s^−1^, LEDs lighting sources) had no significant effect in tatsoi, beet, and parsley microgreens, while it resulted in increased ascorbic acid content in microgreens of amaranth, pea, kale, broccoli, and mustard (by 79.5, 65.2, 60.6, 59.1, and 25%, respectively), and in a decreased content in basil and borage ones (by 53.9 and 46.9%, respectively) [47]. When considering the Chinese kale (*B. oleracea* var. alboglabra Bailey cv. DSCH), a 24 h-red light irradiation (660 nm, 80 µmol m^−2^ s^−1^, LEDs lighting sources) in pre-harvest was effective in enhancing the vitamin C content up to two days of storage after harvesting [140]. A 4-day treatment of mature broccoli heads with red LED light (660 nm) was also effective in reducing the ascorbate loss occurring after the harvest [142], a very desirable result in the light of the high degradation rate of this vitamin.

The limited data available on light quality influence on glucosinolates show that the effect is highly dependent on the wavebands and the plant species. Under red irradiation (730 and 640 nm) sinigrin content of kale was higher as compared to plants grown under blue light [139]. Similarly, when three Chinese cabbage varieties were exposed for 24 h to fluorescent light supplemented with red LEDs (625 nm), the content of total glucosinolates increased in the variety characterised by a low content of these metabolites, while the variety with high glucosinolates positively reacted to supplemental blue radiation [151]. These authors also reported that different set of genes involved in glucosinolates biosynthesis were upregulated by red or blue radiations in Chinese cabbage. To confirm the genotype dependence of the light influence on glucosinolates biosynthesis, Qian et al. [141] did not observe any variation in the content of these compounds in Chinese kale sprouts exposed to red LED light.

## 6. Green Light

Green light, among the whole solar spectrum reaching the Earth’s surface, was considered of less importance in the past, since it was a common belief that it did not affect plants’ growth and development. It has been instead observed that plants reflect just 10–50% of green light [157], contributing to the green appearance of most plant organisms, while the remaining part is mainly absorbed by cryptochromes and by a putative, yet uncharacterised, green-light photoreceptor, and weakly by chlorophylls [20,158,159]. By consequence, green light plays several key roles during plant lifespan, e.g., the shade avoidance responses across the bottom layers of the canopies [158,160]. The LED technology, which is progressively replacing the conventional greenhouse lighting that mostly relies on high-pressure sodium lamps or fluorescent tubes, has allowed researchers worldwide to deepen the knowledge on individual wavelengths, which were previously less considered, e.g., the green light. However, few studies have investigated the effects of green light supplementation on the biosynthesis of bioactive compounds in crops so far (Table 2); therefore, this section will include the most recent literature in the field, without splitting phenolics, terpenoids, and other secondary metabolites.

The tea yellow-leaf mutant plants (O. Kuntze ‘Zhonghuang 3’ (ZH3)) irradiated during the dark period with supplemental green light (520 nm, 300 μmol m^−2^ s^−1^, LEDs lighting sources) for 4 h daily up to 12 days showed enhanced concentration of procyanidin B2/B3, and L-ascorbate [161]. However, when the green light was applied together with the blue light, the increase in secondary metabolites (especially anthocyanins and catechins) was more pronounced than when the green light was applied alone, mainly due to the activation of structural genes of the phenylpropanoid pathway.

When lettuce (var. youmaicai) was grown by cutting out the green light (480–560 nm), the content of photosynthetic pigments and the chlorophylls/carotenoids ratio were reduced, consequently decreasing the CO_2_ assimilation and the growth of the plants [162]. Similar to these findings, another study on lettuce (cv. Butterhead) [163] showed that supplementation of green light (200 μmol m^−2^ s^−1^, LEDs lighting sources) to a 48-h continuous blue and red lighting resulted in increasing the chlorophyll content by inducing an overexpression of photosynthetic genes *LHCb* and *PsbA*; thus, enhancing the photosynthetic rates and the maximal photosynthetic capacity. The positive role of green light in stimulating the accumulation of photosynthetic pigments was also observed in tomato plants (cv. ‘Komeett’) irradiated with 7, 20, or 39% of green light (531 ± 19 nm, 171 μmol m^−2^ s^−1^, LEDs lighting sources) [164]. The authors found an increased chlorophyll *a*/*b* ratio and carotenoids content in the middle leaf layer of the canopy together with the increase of the percentage of green light provided.

Very few studies have investigated the effects of green light irradiation on other secondary metabolites, e.g., phenolic compounds. A comparative study on two basil cultivars, a green leaf (cv. ‘Improved Genovese Compact’), and a purple leaf (cv. ‘Red Rubin’) one, irradiated with increasing proportions of supplemental green light (220 ± 10 μmol m^−2^ s^−1^) resulted in a progressively greater decrease of several bioactive compounds, e.g., phenolics, flavonoids, and anthocyanins [165]. Considering the importance of the genetic background in driving the metabolic responses to the different light radiations, such a negative influence of green light on these metabolites needs to be confirmed in other species and cultivars.

**Table 2:** Biochemical responses of crops and plants of food interest to green light wavelengths considered in this review. Tot, total phenolics; Flav, flavonoids; Ant, anthocyanins; AC, antioxidant capacity; T, terpenoids; AA, ascorbic acid; TP, tocopherols; GSL, glucosinolates. For each plant species and cultivar, and for each secondary metabolite or metabolic class considered, the symbols “↓”, “↑” and “=” mean a decrease, increase or no variations, respectively, compared to the control plants of each study.

Species	Cultivar	Phenolics			AC	T	AA	TP	GSL	Ref.
		Tot	Flav	Ant						
Lettuce (*Lactuca sativa.* L.)	Youmaicai					↑				[162]
	Butterhead					↑				[163]
Tea leaves (*Camellia sinensis* L.O. Kuntze)	Zhonghuang 3		↑				↑			[161]
Tomato plants (*Solanum lycopersicum* L.)	Komeett’					=/↑				[164]
Basil (*Ocimum basilicum* L.)	Improved Genovese Compact	=/↓	=/↓	=/↓	=/↓					[165]
	Red Rubin	=/↓	=/↓	=/↓	=/↓					

## 7. Blue Light

While effects of red and far-red light on phytochemicals accumulation are strictly dependent on the plant species considered, blue light irradiation has been generally reported to enhance the content of most nutraceutical substances, especially in terms of phenolic compounds. However, genotype- and structure-dependent specificity of response was observed as well, as commented in this specific paragraph and depicted in Table 3.

### 7.1. Phenolics

In buckwheat sprouts cultivated in the dark or under blue, red, or fluorescent light, the highest content of total phenolic compounds and total flavonoids was detected following irradiation with blue light (460 nm, 16 h a day, 7 days). An overall increase of the six individual flavonoids resolved by HPLC analysis was observed in blue-irradiated sprouts, their content being about 1.6- to 2.9-fold higher than in dark-grown sprouts [128]. Similarly, soybean (*Glycine max* L. cv. “Dongnong 690”) microgreens exposed for 2 or 4 days to blue light LEDs (450 nm) with a 12 h/12 h (light/dark) photoperiod had higher phenolic content than dark- and white light-grown seedlings. Flavonoid content was instead lowered by 2-day irradiation with blue light, which was, however, effective in increasing this metabolic class when lightning was prolonged up to 4 days [166]. A detailed HPLC–MS analysis highlighted differences in the profile of phenolic compounds, with increased abundance of 6,7,3,4-tetrahydroxyisoflavone, galangin and apigenin 7-O-glucoside and decreased content of dihydrodaidzein 7-O-glucuronide. These changes overall led to enhanced antioxidant activity of seedlings grown under blue light [166].

**Table 3:** Biochemical responses of crops and plants of food interest to blue light wavelengths considered in this review. Tot, total phenolics; Flav, flavonoids; Ant, anthocyanins; AC, antioxidant capacity; T, terpenoids; AA, ascorbic acid; TP, tocopherols; GSL, glucosinolates. For each plant species and cultivar, and for each secondary metabolite or metabolic class considered, the symbols “↓”, “↑” and “=” mean a decrease, increase or no variations, respectively, compared to the control plants of each study.

Species	Cultivar	Phenolics			AC	T	AA	TP	GSL	Ref.
		Tot	Flav	Ant.						
Green leafy lettuce (*Lactuca sativa*. L.)	Thumper	↓		=			↓/↑	↑		[133]
							↑			[167]
	Grizzly						↑			[168]
Red leafy lettuce (*Lactuca sativa.* L.)	Red Cross	=		↑						[131]
Red clover (*Trifolium pratense* L.)						↑				[144]
Chinese cabbage (*Brassica campestris* L.)							↑			[169]
Mustard (*Brassica juncea* L.)	Red Lion					↑		↑		[170]
Beet (*Beta vulgaris* L.)	Bulls Blood					↑		↑		
Parsley (*Petroselinum crispum* Mill.)	Plain Leaved or French					↑		↑		
Buckwheat (*Fagopyrum esculentum*)	Möench	↑	↑							[128]
Wheat (*Triticum aestivum* L.)			↑/↓							[145]
Soybean (*Glycine max* L.)	Dongnong 690	↑	↓							[166]
Bilberry fruit (*Vaccinium myrtillus* L.)				↑						[148]
Apple fruit (*Malus domestica* Borkh.)	Mishima Fuji			↑						[171]
	Jonathan			↑						
Strawberry (*Fragaria × Ananassa*)				↑						[172,173]
	Fengguang						↑			[174]
Cowpea (*Vigna unguiculata* L. Walp.)						↓/↑/=				[147]
Tartary buckwheat (*Fagopyrum tataricum* Gaertn.)						↓				[146]
Pak choi (*Brassica rapa* ssp. chinensis)						↓/=				[150]
Tomato fruit (*Solanum lycopersicum* L.)	Micro-Tom					↑				[175]
Satsuma mandarin fruit (*Citrus unshiu* Marc.)						↓/↑/=				[152]
Tea leaves (*Camellia sinensis*)	Jinxuan					↑				[149]
Basil (*Ocimum basilicum* L.)	Genovese					↑				[176]
Satsuma mandarin fruit (*Citrus unshiu* Marc.)							↑			[177]
Valencia orange fruit (*Citrus sinensis* Osbeck)							↑			
Lisbon lemon fruit (*Citrus limon* Burm.f.)							↑			
Canola (*Brassica napus* L.)									↑/=	[178]
Mustard (*Brassica juncea* L.)								↓		[179]

A structure-dependent response to blue radiation (470 nm) was also observed in wheat sprouts irradiated for up to 12 days (a 16-h light/8-h dark photoperiod). This treatment led in fact to a decreased accumulation of p-coumaric acid and epicatechin while gallic acid and quercetin content increased in comparison to sprouts irradiated with white light [145]. It should be noted that what the authors call “white light” is a treatment with 380 nm radiation, i.e., long wave UV-A radiation.

On the contrary, adding blue light LEDs (455, 470 nm, 30 μmol m^−2^ s^−1^) to standard high-pressure sodium (HPS) lamps in Romaine green baby leaf lettuce (cv. Thumper) determined a decrease in total phenols concentration compared to the HPS alone, while no effects in anthocyanin content was registered [133]. Cultivar and treatments, however, might result in completely different responses in terms of phenolics accumulation. Indeed, a 12-day blue light supplementation (476 nm, 130 ± 10 μmol m^−2^ s^−1^, LEDs lighting sources) on baby leaf lettuce (cv, Red Cross) was effective in stimulating anthocyanin accumulation without increasing the total phenolic content [131]

Blue light was generally reported to stimulate anthocyanin biosynthesis, resulting in marked accumulation of these metabolites, particularly in fruits. In bilberries, 48-h irradiation with blue light (400–500 nm, 8.10 μmol m^−2^ s^−1^) was effective in inducing anthocyanins accumulation over the control fruits grown under white light [148]. However, the effect induced by blue light on total anthocyanin and delphinidins content did not differ markedly from that induced by red or far-red irradiation. Moreover, when considering the other subclasses, cyanidins, peonidins, and malvidins were unaffected, while petudinins increased, but less than under red and far-red lightning.

Irradiation for 96 h with blue LEDs (430, 450, 470, and 490 nm) of apple (*Malus domestica* Borkh.) fruits harvested at the mature green stage induced an increased accumulation of anthocyanins and the development of the red colour, in both “Mishima Fuji” and “Jonathan” cultivars, though, because of the different genetic background, Jonathan cv accumulated more anthocyanins that Fuji under blue light [171]. The involvement of the blue light photoreceptor cryptochrome of apple in promoting anthocyanin accumulation was demonstrated using *MdCRY2* transgenic Arabidopsis [180]. Moreover, CRY1, CRY2, and CRY3, and PHOT1 and PHOT2 were all downregulated in strawberry fruits treated with blue light [172] and a decreased transcription of photoreceptor genes, except PHOT2, occurred during fruit development from green to red ripe stage, suggesting a role of PHOT2 in blue light-induced anthocyanin accumulation [181]. Indeed, total anthocyanins, as well as the individual anthocyanins pelargonidin 3-glucoside (accounting for more than 80% of total anthocyanins) and pelargonidin 3-malonylglucoside, were more concentrated in strawberries ripened *in planta* under blue (450 nm, 8 h dark–16 h light photoperiod) than under white light [172]. Blue light irradiation (40 μmol m^−2^ s^−1^ for up to 12 days at 5 °C) was also effective in improving total anthocyanin content of strawberries when applied in post-harvest [173], suggesting that supplemental blue light during storage could be helpful in preserving or even improve the quality of post-harvest fruits.

### 7.2. Terpenoids and Chlorophylls

The effect of blue radiation on carotenoids was a little bit more variable than on phenolic compounds. A 7-day supplementation of blue light (440 nm, 150 ± 5 μmol m^−2^ s^−1^, LEDs lighting sources) on red clover sprouts was effective in significantly enhancing the concentration of the main carotenoids (β-carotene, lutein, and zeaxanthin) compared to irradiation with only white light [144]. Similarly, cowpea sprouts grown under blue LEDs irradiation (16 h photoperiod, 470 nm, 50 μmol m^−2^ s^−1^) contained the highest levels of total carotenoids and of lutein, 13Z-β-carotene, and E-β-carotene as compared to sprouts grown under white, red, or blue–red mix. Moreover, α-carotene and 9Z-β-carotene level was higher than in sprouts cultivated under red- and blue–red mixed radiation, and unchanged in comparison to white light treated samples, while zeaxanthin was decreased by this light radiation [147]. An opposite behaviour was shown in tartary buckwheat sprouts grown under blue LEDS (470 nm, 50 μmol s^−1^ m^−2^, 16 h photoperiod), where total carotenoids, as well as individual xanthophylls and carotenes, except for zeaxanthin, were less concentrated than in samples grown under white light [146].

When considering microgreens, blue light (445 nm, 300 ± 3 μmol m^−2^ s^−1^, LED lighting sources) supplementation (+33% to the standard light conditions) determined an increased content of several carotenoids (α- and β-carotenes, lutein, violaxanthin, and zeaxanthin) ranging from 1.2 to 4.3 times in mustard, beet, and parsley [170]. A structure-specific effect of blue LED (peak at 453 nm) irradiation was observed in pak choi sprouts that exhibited a lower concentration of lutein and total carotenoids, while β-carotene and violanthin were unaffected by this radiation [150].

Blue light irradiation (12 days, 476 nm, 130 ± 10 μmol m^−2^ s^−1^) increased the concentration of total xanthophylls and β-carotene also in baby leaf lettuce (cv. Red Cross) [131], while a decreased accumulation of both α- and β-carotenes was observed following the addition of blue light (445 or 470 nm, 30 μmol m^−2^ s^−1^, LEDs lighting sources) during cultivation of Romaine baby leaf lettuce [133].

Carotenoid content can be modified by blue radiations also in fruits, as shown by the research carried out by Xie et al. [175] on tomato fruit (cv. Micro-Tom) ripened in planta under supplemental blue light (430 nm, 12 h photoperiod). These authors detected higher concentrations of lycopene in fruits exposed to blue light supplementation when compared to natural light conditions and red light (660 nm) supplementation at 42, 48, and 54 DAA (days after anthesis), and of β-carotene at 48 and 54 DAA, while lutein was more concentrated than in control fruits only at 36 DAA. Involvement of HY5 in mediating the increased transcription of *PSY1* (*phytoene synthase 1*) gene, the key limiting step for carotenoid synthesis in tomato ripening fruit [182], was proposed [175].

An unchanged level of total carotenoids was observed in the flavedo of Satsuma mandarin fruits irradiated in post-harvest irradiated with blue (470 nm) LEDs (50 μmol m^−2^ s^−1^) for 6 days. This apparent absence of blue light-induced effects was indeed due to different trends of variations experienced by the individual carotenoids present in this fruit as compared by dark-treated ones. Specifically, the content of α- and β-carotene, lutein, and all-*trans*-violaxanthin increased, while all-*cis*-violaxanthin decreased and β-cryptoxanthin was unaffected [152], highlighting once again the complexity of modulation of the secondary metabolism by specific light wavelengths.

As already reported for red light treatment, a 3-day exposure to blue light (470 nm, 70–80 μmol m^−2^ s^−1^) induced increased production of some volatile terpenes in tea leaves. Geraniol, linalool, linalool oxide, and diendiol I were all produced at higher concentration than in dark-treated leaves and linalool and diendiol I reached the highest concentration also when compared to leaves exposed to red light [149]. Cultivation of basil with supplemental LED treatments with progressive blue/red ratios (447 nm/627 nm, from 10/90 to 60/40 blue/red) increased the concentration of eucalyptol, linalool, (R)-(+)- and (S)-(−)- limonene, and α- and β-pinene in comparison to natural light controls [176], indicating the great potential to influence the production of volatile molecules and, consequently the flavour quality of beverage plants and herbs, by manipulating the growth light environment.

This portion of the light spectrum is able to modulate chlorophyll content as well. An increased content of both chlorophyll *a* and *b* in mustard, beet, and parsley microgreens was induced by blue light supplementation [170]. However, chlorophyll content of baby leaf lettuce (cv, Red Cross) irradiated by blue light (12 days, 476 nm, 130 ± 10 μmol m^−2^ s^−1^) was unchanged [131], while in pak choi sprouts cultivated under blue LEDs (peak at 453 nm) chlorophyll *b* (but not chlorophyll *a*) concentration was lower than in control sprouts grown under white light [150]. As for the other metabolites, a genotype-specific response to blue radiation seems to occur also for chlorophylls.

### 7.3. Other Secondary Metabolites

The influence of increasing dosage of blue light (from 0 to 33%, 445 nm) to the LED-based lighting conditions composed by a mix of 638 + 660 + 731 nm (total PPFD 300 ± 3 μmol m^−2^ s^−1^) on tocopherols concentration was investigated in mustard, beet, and parsley microgreens [170]. In all species, the best effect on total tocopherols level was induced by a 16% blue light enrichment, because of a significant enhancement of specific compounds in the different microgreens: α- and β-tocopherol in mustard, γ- and δ-tocopherol in beet and β-, γ-, and δ-tocopherol in parsley [170]. Increasing of blue light to 33% further increased total tocopherols in beet, but not in mustard and parsley.

Similarly, a positive effect of blue light LEDs (455 and 470 nm, 30 μmol m^−2^ s^−1^) was observed in Romaine green baby leaf lettuce cv. Thumper, where a higher α- and γ-tocopherols content were detected as compared to HPS alone [133].

The current literature reports evidence that the blue portion of the light spectrum may modify the concentration of ascorbic acid as well, though it was not a general effect, but it depended on the plant species or cultivar considered, and/or the specific wavelength. In Romaine green baby leaf lettuce (cv. Thumper) the content of ascorbic acid showed indeed an opposite response to supplemental irradiation with 455 or 470 nm blue light, specifically, a decrease or an increase respectively [133]. Considering the Red Cross cultivar, however, ascorbic acid content was unaffected after a 12-day blue light supplementation (476 nm, 130 ± 10 μmol m^−2^ s^−1^, LEDs lighting sources) [131].

However, most studies highlighted the ability of blue light to enhance ascorbate levels. Zha et al. [167] observed a positive influence of increased proportion of blue light on ascorbate concentration in lettuce cultivated with different red/blue light ratios (75/25, 50/50, 25/75 R/B) for 12 days (24 h a day, 200 μmol m^−2^ s^−1^). Such an increase was accompanied by a transient overexpression of many genes involved in ascorbate biosynthesis, and a more consistent increase in the activity of enzymes involved in ascorbate regeneration, leading the authors to conclude that the higher levels of ascorbate observed in the 25/75 R/B growth condition were the consequence of a better regeneration activity rather than enhanced biosynthesis under blue light. Similarly, vitamin C concentration was 2.25-fold higher in lettuce (cv. Grizzly) under 100% blue light irradiation (460–475 nm, 14 h photoperiod, 300 μmol m^−2^ s^−1^) as compared to ambient light [168]. A positive influence of blue radiation was also observed in non-heading Chinese cabbage seedlings, where the concentration of ascorbate was highest under blue/red mixed irradiation (11.1/88.9 ratio) followed by 100% blue lightening [169].

Irradiation of the juice sacs of Satsuma mandarin (*Citrus unshiu* Marc.), Valencia orange (*C. sinensis* Osbeck), and Lisbon lemon (*C. limon* Burm. f.) with blue LEDs (470 nm) was effective in increasing the ascorbate content in comparison to the dark- and red-light exposed samples after both 2 and 4 weeks of treatment [177]. Interestingly, continuous lightening was more effective than pulsed irradiation in all three species. Moreover, post-harvest blue light irradiation (470 nm, 40 μmol m^−2^ s^−1^) promoted higher accumulation of vitamin C in strawberry fruits in comparison to dark-stored fruits [174].

A positive influence of blue radiation (470 nm, 16 h photoperiod, 50 μmol m^−2^ s^−1^ for 14 days) on glucosinolates was detected in canola (*Brassica napus* L.) sprouts [178]. Although the total content did not differ among sprouts grown under blue, red, or white light, some specific individual glucosinolates were highly accumulated following blue irradiation. Specifically, these sprouts contained the highest levels of glucoraphanin, and shared the primacy with white light-grown sprouts relative to glucoalyssin and gluconapin, and with red light-grown sprouts relative to progoitrin and neoglucobrassicin. Conversely, blue light led to the lowest levels of sinigrin and glucobrassicin. Park et al. [179] investigated the influence of blue light (450 nm, 16 h photoperiod, 90 μmol m^−2^ s^−1^) on glucosinolate content of *Brassica juncea* sprouts cultivated for up to 3 weeks. Sprouts grown under blue radiation had the lowest content of total glucosinolates, independently on the growth period (1, 2, or 3 weeks). Only glucoiberin and gluconasturtiin were unchanged as compared to both red and white light treatments, while, generally, the other specific molecules were less concentrated. A species-specific, as well as a structure-dependent influence of blue radiation on these bioactive molecules, is evident.

## 8. UV-A Radiation

The physiological and biochemical effects of UV-A radiation are strictly dependent on both the plant species and the UV-A dose. Endemic plants (and crops) from high altitude areas and/or low latitude regions are well acclimated to high UV (-A and -B) condition and, therefore, care must be given when establishing the UV dose needed to stimulate their secondary metabolism further. Table 4 lists some main biochemical responses observed in different plant species or cultivars subjected to UV-A irradiation, as detailed in the following paragraph.

### 8.1. Phenolics

Like blue light, most relevant researches carried out by supplying or depriving the plants of UV-A observed a general positive effect of this radiation in stimulating the accumulation of health-promoting flavonoids in many species. Irradiation of a red- and a green-leaf cultivar of pak choi with 12-h daily UV-A (380 nm, 100 μmol m^−2^ s^−1^, LEDs lighting sources) for 10 days resulted in increased content of total phenolics, flavonoids, and anthocyanins in the red cultivar, whereas only anthocyanins were enhanced in the green cultivar [183]. However, when UV-A wavelength was 400 nm, the green leaf variety positively responded in terms of total phenolics and flavonoids. Moreover, antioxidant capacity of both cultivars significantly increased regardless the UV-A wavelength used [183]. The discrepancy of responses in terms of phenolics and flavonoids is most likely due to the genetic predisposition of the red cultivar, over the green cultivar, to naturally synthesize and accumulate phenolic compounds in the leaves.

Positive effect of UV-A exposure (320–400 nm, 3.0 W m^−2^, 24 h, fluorescent lamp as lighting source) in enhancing the anthocyanin content was observed also in turnip seedlings (*Brassica rapa* subsp. *rapa*, cv. Tsuda) [184]. Another work on 7-day-old broccoli sprouts (*Brassica oleracea* L., var. italica, cv. Waltham 29) exposed for 120 min to either 3.16 (low dose) or 4.05 (high dose) W m^−2^ UV-A radiation (UV-A lamp as lighting source) found structure-dependent responses among the 22 phenolic compounds identified [164]. Moreover, the low dose of UV-A was more effective than the high dose in stimulating the phenolics accumulation, particularly gallic acid hexoside, 4-O-caffeoylquinic acid, gallic acid derivative, and 1-sinapoyl-2,2-diferulolyl-gentiobiose, when plants were harvested 2 h after the UV treatment [185]. Another study on a different cultivar of broccoli (cv. Monopoly) exposed to two different UV-A conditions (365 nm, 61 ± 3 μmol m^−2^ s^−1^; 385 nm, 15 ± 3 μmol m^−2^ s^−1^, LEDs lighting sources) found that the shortest UV-A wavelength (365 nm) induced a significant reduction of all the hydroxycinnamic acids identified, while the 385 nm UV-A irradiation had no effect [186]. Moreover, the 365 nm UV-A exposure determined a structure-dependent response by quercetin and kaempferol glycosides since several of them decreased, while others were unaffected by the treatment. Contrarily, the longest UV-A wavelength did not induce almost any variation in the level of these compounds [186]. Such a specificity of response by the individual flavonoids was observed also in Brussels sprout plants (*B. oleracea* var. gemmifera DC) exposed to UV-A radiation (365 nm), that underwent a decrease in the concentrations of sinapic acid acylated kaempferol tri- and tetraglycosides, while kaempferol-3-O-disinapoyl-triglucoside-7-diglucoside accumulated at higher level than in control samples [187].

**Table 4:** Biochemical responses of crops and plants of food interest to UV-A wavelengths considered in this review. Tot, total phenolics; Flav, flavonoids; Ant, anthocyanins; AC, antioxidant capacity; T, terpenoids; AA, ascorbic acid; TP, tocopherols; GSL, glucosinolates. For each plant species and cultivar, and for each secondary metabolite or metabolic class considered, the symbols “↓”, “↑” and “=” mean a decrease, increase or no variations, respectively, compared to the control plants of each study.

Species	Cultivar	Phenolics			AC	T	AA	TP	GSL	Ref.
		Tot	Flav	Ant						
Pak-choi (*Brassica rapa* ssp. chinensis var. communis)	Red leaf cv.	↑/=	↑	↑	↑	↑/=	↑/↓	↑	↑	[183,188]
	Green leaf cv.	↑/=	↑/=	↑	↑	=	↓		↑	
Turnip (*Brassica rapa* subsp. *rapa*)	Tsuda			↑						[184]
Broccoli (*Brassica oleracea* L., var. italica)	Waltham 29	=/↓							=/↓	[185]
	Monopoly	=/↓							=	[186]
Broccoli (*Brassica oleracea* L., var. gemmifera DC)		↑/↓							↑/=	[187]
Lettuce (*Lactuca sativa.* L.)	Yanzhi	=	↑	↑	↑	↓	↑			[189]
	Red butter	↑	↑	↑	=	↓	↑			
	Klee	↑/=	↑/=	↑			↑			[190]
	Red leaf cvs.					=				[191]
	Green leaf cvs.					=				
	Hongyeom	↑/=		↑/=	↑/=					[192]
Tomato plant (*Solanum lycopersicum* L.)	Oxheart	↓		=	=	=				[193]
	Cherry	=		↓	↓	↓/=				
	Roma	=		=	↓	↑/=				
	MicroTom			↑						[194]
Tomato fruit (*Solanum lycopersicum* L.)	Budenovka	↑	↑			↑/=				[195]
	Bull Heart	↑	↑			↑/=				
	Gina	↑	↑			↑/=				
	Micro-Tom			↑						[194]
Sowthistle (*Ixeris dentata* Nakai)		↑/=	↑/=		↑/=					[196]
Grape berry (*Vitis vinifera* L.)	Cabernet Sauvignon		↑							[197]
Blueberry (*Vaccinium corymbosum* L.)	Duke	↓		=						[198]
Peach fruit (*Prunus persica* L. Batsch)	Hujingmilu			↑						[199]
	Yulu			=						
Basil (*Ocimum basilicum* L.)	Genovese	↑/=				↑	↓	↑/↓		[188,200,201,202,203]
Beet (*Beta vulgaris* L.)	Bulls Blood						↑/↓	↑		[188]
Rice (*Oryza sativa* L.)	Kanchana	↑				↑				[204]
	Mattatriveni					↓/=				
	Harsha					↑/=				
Broccoli (*Brassica oleracea* L. var. *italica*)	Waltham 29	=							↑	[205]
Wheat (*Triticum aestivum* L.)	Sumai188	↑								[206]
Mung bean (*Vigna radiata*)		↑/↓	↑				↑			[207]
Peppermint (*Mentha piperita* L.)	Rubescens	↑				↑/↓				[208]

A comparative study on two lettuce cultivars (cvs. Yanzhi and Red butter) irradiated with UV-A (380 ± 10 nm, 10 μmol m^−2^ s^−1^, LEDs lighting sources) found that the treatment positively affected the content of total polyphenols, flavonoids, and anthocyanins, increasing the antioxidant activity (using the 2,2-diphenyl-1-picrylhydrazyl (DPPH) assay) in Yanzhi cultivar, leading to a higher nutraceutical value of the UV-A-treated plants [189]. The same positive results were also obtained in the red-leaf lettuce cv. Hongyeom irradiated for 7 days continuously (352 nm, 3.7 W m^−2^, fluorescent lamp as lighting source) in enhancing the anthocyanin content (fluorescent lamps as a lighting source was observed) that underwent a transient enhancement of total phenolic and anthocyanin concentration, as well as antioxidant capacity, within the first 2–3 days of treatment [192]. Other research carried out on lettuce (cv. Klee) treated with three different UV-A doses (365 nm; 10, 20, 30 μmol m^−2^ s^−1^, LEDs lighting sources) confirmed the positive influence of UV-A radiation on phenolic compounds and highlighted a dose-response effect. Specifically, the highest dose was effective in enhancing the total phenolic, flavonoid, and anthocyanin content, the intermediate dose increased the total flavonoid and phenolic content, while the lowest dose stimulated only the accumulation of anthocyanins [190], suggesting the high sensitivity of this flavonoid class to UV-A radiation.

In contrast to the results above, treating tomato seedlings of three different cultivars (*Solanum lycopersicum* L., cvs. Oxheart, Cherry, and Roma) with 2 h daily of UV-A (368 nm, 0.45 W m^−2^, UV-A blacklight lamps as lighting source) resulted in decreasing the anthocyanin content in cv. Cherry, while no changes were observed for the other two cultivars [193]. In addition, total phenolic content was not affected by the treatment in cvs. Roma and Cherry, whereas a decrease was observed in cv. Oxheart, while antioxidant activity decreased in cvs. Cherry and Roma. Contrasting results were obtained irradiating tomato seedlings (*Solanum lycopersicum* L. cv. MicroTom) with 24-h UV-A (365 nm, 7 W m^−2^, fluorescent tubes as lighting sources) [194]. Indeed, a significant accumulation of anthocyanins were observed whether both the seedlings were exposed to only UV-A or a combination of visible light + UV-A. Moreover, such significant anthocyanin accumulation started from 1-h irradiation in the cotyledon, and started from 3 h in the hypocotyl, reaching the maximum level after 12 h of UV-A exposure. In sowthistle plants (*Ixeris dentata* Nakai), a 7-day, 24-h continuously UV-A exposure (352 nm, no UV-A dose specified, fluorescent tubes as lighting source) determined a transient increase in phenolics concentration and antioxidant activity after 3 days from the beginning, together with a transient increase in flavonoid content after 5 days [196].

Some research on the influence played by UV-A on fruit phenolics were also published. Generally, the effect was positive, leading to higher accumulation of total phenolics and flavonoids in irradiated fruits, though with differences ascribable to genotype and wavelength. Specifically, three tomato cvs (Budenovka, Bull Heart, and Gina) irradiated at the red ripe stage with UV-A lamps emitting 353, 365, and 400 nm (irradiance 0.33, 0.28 and 0.28 W m^−2^, respectively) for 10, 180, or 360 min showed an increased concentration of phenolics and flavonoids, starting from 180 min of irradiation and reaching the maximum levels at the highest exposure time. Independently from the duration of the treatment, irradiation with 365 nm induced the highest phenolic and flavonoid accumulation in all the cvs, while some differences among the cvs were observed in the phenolic reaction to the other wavelengths. However, after 360 min of irradiation, no difference among the three wavelengths was detected, with the only exception of flavonoid content of cv. Budenovka, which was unchanged following the irradiation with the shortest wavelength [195]. Grape berries (*Vitis vinifera* L. cv. Cabernet Sauvignon) harvested at different developmental stages and exposed to UV-A radiation for 20 min (total dose of 1.8 kJ m^−2^, corresponding to 1.5 W m^−2^) accumulated higher flavan-3-ols than control and showed increased transcription of three biosynthetic genes [197]. Increased anthocyanin accumulation was observed in the peel of the peach (*Prunus persica* L. Batsch) cv. Hujingmilu, but not in cv. Yulu, irradiated in post-harvest with UV-A (315–400 nm, 10 W m^−2^) for 2 days before turning stage. Such an accumulation (four-fold higher than in control) was accompanied by an evident reddening of the fruit peel [199]. Similarly, UV-A irradiation (365 nm, 7 W m^−2^) of tomato fruits (cv Micro-Tom) was successful in promoting a significant increment in anthocyanin content, which reached a maximum after 6 h of irradiation [194]. However, post-harvest irradiation of blueberries (*Vaccinium corymbosum* L.) with 10 W m^−2^ UV-A (λ_max_ 352 nm) for 10 min on the top and 10 min on the bottom side of the fruits did not induce any increase in anthocyanin accumulation during the 28-day storage period, and even led to a slight transient decrease of total phenolic content [198]. Despite UV-A was generally found to play a positive influence on phenolic biosynthesis, interaction among genotype, radiation wavelength, and duration of exposure is ultimately responsible of the observed response. Therefore, establishing the most adequate irradiation protocol for any specific species or cultivar is mandatory to obtain the desired improvement of the nutritional quality.

### 8.2. Terpenoids and Chlorophylls

While influence of visible light on carotenoids is expected, due to their spectrum of absorbance, the impact of UV radiation on these compounds is less obvious. Similar to what reported for blue light, UV-A seems to affect carotenoids in a very variable way, depending on specific compound, plant species, organ, UV-A wavelength, and dose. However, the few examples reported below suggest that UV-A is not so efficient in inducing carotenoid overproduction. Such a positive result was observed only with long UV-A wavelengths (400 nm), at the border with blue portion of the solar spectrum. Indeed, in pak choi plants, the red-leaf cultivar underwent a significant increase in carotenoid content when irradiated with 400 nm UV-A (100 μmol m^−2^ s^−1^, LEDs lighting sources), but not with 380 nm UV-A at the same irradiance. Moreover, no variations were observed in the green cultivar for any of the UV-A wavelength considered [183].

Two research studies were recently published, reporting different effects induced by UV-A radiation on lettuce carotenoids. Specifically, UV-A treatment (380 ± 10 nm, 10 μmol m^−2^ s^−1^, LEDs lighting sources) led to a significant decrease in carotenoid content in cvs. Yanzhi and Red butter [189]. Contrarily, another lettuce cultivar (cv. Klee) exposed to three different UV-A doses (365 nm; 10, 20, 30 μmol m^−2^ s^−1^, LEDs lighting sources), did not show any variation in terms of total carotenoid content [190]. Because of the different wavelengths used in the two experiments, the behaviour exhibited by the three cultivars cannot be univocally attributed to genotype. This is a common problem found when comparing results obtained by applying different irradiation protocols.

A comprehensive study on eight red lettuce varieties (cvs.: Black Jack, Galactic, Impuls, Dark Lollo Rossa, New Red Fire, Rave, Red Sails, and Vulcan) and eight green lettuce varieties (cvs.: Black-Seeded Simpson, Concept, Crisp and Green, Envy, Marin, Simpson Elite, Two Star, and Waldmann’s Dark Green) exposed to supplemental UV-A radiation (320–400 nm) clearly showed that no variety, regardless of the leaf colours, was affected by the treatment [191]. Unfortunately, however, due to the lack of information on the UV-A exposure conditions, it is impossible to state whether the undetected changes in carotenoid accumulation were due to an insufficient UV-A irradiation or other reasons.

A confirmation of the incapacity of UV-A to increase the leaf carotenoid content derives from a study carried out by exposing seedlings of three tomato cultivars (*Solanum lycopersicum* L., cvs. Oxheart, Cherry, and Roma) to UV-A radiation (2 h daily treatment, 368 nm, 0.45 W m^−2^, UV-A blacklight lamps as lighting source). In fact, carotenoids concentration was unaffected in cv. Oxheart and Roma and even decreased in cv. Cherry [193]. This work also clearly underlines the diversity of response towards UV-A radiation within the same species, but among different cultivars.

Differently, in tomato fruits, UV-A irradiation for 360 min with 365 and 400 nm led to a significant increase in total carotenoids level in all the three cvs. studied (Budenovka, Bull Heart, and Gina). However, 353 nm was never effective in inducing modification in carotenoid content, while 365 nm played a positive influence on carotenoid levels already after 10 min of irradiation, but only in cv Budenovka [195], again indicating the importance of both light energy and genotype in determining the outcome. The same authors also found a structure-dependent response to the three UV-A radiations employed in their research. Indeed, for any cultivar, while β-carotene, lycopene, lutein increased starting from 180 min of irradiation with 365 nm, after 360 min of irradiation the longest wavelength was effective only for β-carotene and lutein.

UV-A radiation had an impact also on chlorophylls, as observed in two lettuce cultivars (Yanzhi and Red butter) exposed to 10 μmol m^−2^ s^−1^ (380 nm) that exhibited an increased content of these pigments [189]. However, no change in chlorophyll *a* + *b* concentration was reported in cv. Klee following irradiation with three different UV-A doses (365 nm; 10, 20, 30 μmol m^−2^ s^−1^ [190]. Similarly, UV-A-dependent accumulation of chlorophylls was induced in the tomato cv. Roma, but not in cv. Oxheart and cv. Cherry, following 2 h daily irradiation with 368 nm (0.45 W m^−2^) [193], due to the different responsiveness to this radiation linked to genotype.

A study carried out in fruits of three tomato cultivars (Budenovka, Bull Heart, and Gina) highlighted the dependence of chlorophyll accumulation on UV-A wavelength. Indeed, 180- and 360-min treatment with 365 nm increased chlorophyll concentration in all cultivars, while the highest wavelength induced a positive effect only after 360-min exposure and only for two of the three cultivars, while 353 nm was always ineffective [195]. A similar finding was also reported by Brazaitytė et al. [188] in red pak choi seedlings grown under supplemental UV-A radiation (366, 390, or 402 nm, the latter already falling into the blue region of the spectrum) given at two different doses (6.2 or 12.4 μmol m^−2^ s^−1^). An increased chlorophyll accumulation was in fact observed after irradiation with any of the three wavelengths at the lowest dose, but only with 390 nm at the highest one.

### 8.3. Other Secondary Metabolites

Effects of UV-A on different classes of metabolites is highly variable in relation to the treatment condition and crop species. The content of α-tocopherol increased in fact in microgreens of beet (*Beta vulgaris* L., cv. Bulls Blood) and red pak choi (cv. Rubi) grown under 12.4 μmol m^−2^ s^−1^ supplemental UV-A radiation (366, 390, or 402 nm, the latter wavelength indicated by the authors as UV-A, though it should be considered already as blue radiation). However, in basil (cv. Sweet Genovese) microgreens, only the shortest wavelength had a positive effect, while the other two led to a reduced α-tocopherol content [188]. Moreover, irradiation with 6.2 μmol m^−2^ s^−1^ UV-A at any wavelength resulted in a significant decrement of α-tocopherol content, except in pak choi under supplemental 390 or 402 nm irradiance. At the lowest UV-A irradiance, the balance between the stressful potential of the radiation and its capacity to stimulate the biosynthetic pathway was, perhaps, unbalanced towards the former.

In the same experiment, a variable influence of UV-A radiation on ascorbic acid was observed, depending on plant species, wavelength, and irradiance level. For example, in basil, the treatment with 366 nm always had a negative outcome, in pak choi—a positive influence, while in beet seedlings, this radiation increased or decreased the level of ascorbic acid when given at the highest or the lowest dose, respectively [188]. When considering lettuce (cvs. Yanzhi and Red butter), irradiation with UV-A (380 ± 10 nm, 10 μmol m^−2^ s^−1^, LEDs lighting sources), resulted in remarkably increasing the vitamin C content in both the cultivar considered [189]. This effect is also confirmed in another lettuce cultivar (Klee), where all three UV-A doses tested (365 nm; 10, 20, 30 μmol m^−2^ s^−1^, LEDs lighting sources) were effective in significantly enhancing the ascorbic acid content [190]. Conversely, when irradiated with 380 and 400 nm UV-A (100 μmol m^−2^ s^−1^, LEDs lighting sources), both green and red cultivars of pak choi underwent a depletion of vitamin C concentration [183].

However, the same treatments led to increased accumulation of glucosinolates, another class of secondary metabolites found to be sensitive to UV-A irradiation. The influence of UV-A on glucosinolates depended on the irradiance level, as reported in a study on broccoli sprouts (cv. Waltham 29), where a low irradiation (3.16 W m^−2^, UV-A lamp as lighting source) determined a decrease in glucoiberin, glucoraphanin, and 4-hydroxy-glucobrassicin, while a high irradiation (4.05 W m^−2^) was effective in increasing these glucosinolates, as well as glucoerucin, glucobrassicin, and 4-methoxy-glucobrassicin compared to the control, when plants were sampled 2 h after the treatment [185]. Moreover, the authors showed the concentration of several glucosinolates changed if plants were sampled 24 h after the irradiation. A difference response in glucosinolates content was observed in another broccoli cultivar (cv. Monopoly) subjected to two UV-A treatments (365 nm, 61 ± 3 μmol m^−2^ s^−1^; 385 nm, 15 ± 3 μmol m^−2^ s^−1^, LEDs lighting sources) [186]. Indeed, both the aliphatic and the indolic glucosinolate were unaffected by UV-A, except 4-methoxy-3-indolylmethyl, which increased after the 365 nm UV-A irradiation. Acharya et al. [187] observed an increased concentration of total indole, but not aliphatic, glucosinolates, in Brussels sprouts grown with supplemental UV-A radiation (365 nm). Moreover, within the indole glucosinolates class, some specific compounds were unaffected, indicating a structure-dependent influence of UV-A radiation.

## 9. UV-B Radiation

Similar to the findings reported for the other wavelengths, the influence of UV-B radiation on the content of bioactive compounds in different species and cultivars is variable and often dependent on the dose and metabolite considered. Examples of these specific responses are reported in the following paragraphs and listed in Table 5.

### 9.1. Phenolics

Most current literature on crop plants agree that UV-B exposure, in dependence on the UV-B-dose applied, triggers the biosynthesis of phenolics compounds, particularly flavonoids. This is mainly due to the biochemical properties of these metabolites, being strong reactive oxygen species (ROS) scavengers and UV-B-absorbers, thus acclimating the plant towards ambient UV-B conditions.

UV-B supplementation (290–320 nm, 14.4 kJ m^−2^ d^−1^, corresponding to 0.17 W m^−2^, broadband UV-B lamp as lighting source) for 4 days was effective at increasing the epidermal flavonoid content of basil plants, both after the treatment and after 7 days of storage. Moreover, concentration of rosmarinic, caffeic, and cichoric acids, catechin derivative, and total hydroxycinnamic acids was higher in leaves of treated plants after the 7-day storage [201]. Another study on basil plants [200] investigated the influence of different UV-B doses (8.5, 34, 68, 102 kJ m^−2^ d^−1^, corresponding to 0.1, 0.4, 0.8, and 1.18 W m^−2^, respectively, broadband UV-B lamp as lighting source) given as acute (a single UV-B irradiation the first day + 3 days of recovery), sub-acute (multiple UV-B irradiations the first day + 3 days of recovery), or sub-chronic (a single UV-B irradiations per day for 6 consecutive days + 3 days of recovery) treatment on phenolic content. The authors found that the acute and sub-acute exposures resulted in increasing phenolics concentration regardless the UV-B dose, especially after 48 and 72 h from the beginning of recovery phase, except for the lowest UV-B dose in the sub-acute treatment. The phenolics accumulation found during the recovery period is somehow expected, due to the time needed to induce gene transcription and the following biochemical rearrangements of the biosynthetic machinery. Indeed, when the sub-chronic treatment, lasting 6 days, was applied, the highest doses (34, 68, and 102 kJ m^−2^ d^−1^, i.e., 0.4, 0.8, and 1.18 W m^−2^, respectively) also induced a phenolics accumulation regardless the recovery time considered but, contrary to the acute and sub-acute irradiations, the enhanced phenolics level was observed already during the irradiation period.

The influence of genotype, UV-B dose, and chemical structure of the target metabolites was evident in a research carried out on green- and red-leaf lettuce (cv. Salad Bowl) plantlets exposed for two weeks to daily UV-B irradiation (1.69 W m^−2^, 1 hr per day, UV-B lamp tubes). In UV-B-treated green plants, quercetin and luteolin glycosides, caffeoyltartaric acid, caffeoylmalic acid and caffeoylquinic acid accumulated at a higher level than in the control lettuce after two weeks of exposure, while only the latter increased after 1 week. The red-leaf lettuce was more responsive to UV-B treatments, showing increased contents of quercetin and luteolin glycosides, as well as caffeoyltartaric acid and caffeoylquinic acid already after one week of exposure. The content of the anthocyanin cyanidin-3-malonylglucoside, present only in red lettuce, also accumulated at higher levels after both one and two weeks of UV-B treatment. However, caffeoylmalic acid was negatively affected by both UV-B doses [209].

A significant increase in total phenolic content regardless the UV-B-dose applied (7, 14, 21, 28 kJ m^−2^ d^−1^ UV_BE_) was detected in rice (*Oryza sativa* L., cv. Kanchana) seedlings grown under supplemental UV-B irradiation for one week continuously (280–320 nm, 300 μmol m^−2^ s^−1^, UV-B lamp as lighting source) [204]. Despite UV-B is frequently reported to stimulate phenolic accumulation, this is not a general rule. One reason could be the structure-dependent influence of this radiation on specific molecules that, leading to an increase of some compounds and to a decrease of some others, ultimately results in an invariance of the total phenolics content. Indeed, 7-day-old broccoli sprouts exposed to 120 min UV-B radiation (7.16 W m^−2^, UV-B broadband lamps as lighting source) did not show any significant variation in total phenolic concentration, although some individual phenolic compounds registered either an increase (gallic acid hexoside II) or a decrease (3-*O*-hexoside kaempferol, 1,2-disinapoyl-2-ferulolylgentiobiose, 5-sinapoylquinic acid, 1,2-diferulolylgentiobiose) [205]. Similarly, in peppermint (*Mentha piperita* L. nm rubescens) plants, grown either in open field and in growth chambers, and exposed to 1-h UV-B radiation a few days after full bloom (310 nm, 7.1 kJ m^−2^ day^−1^ UV_BE_, UV-B broadband lamps as UV-B source), the total phenolic content increased regardless the growing condition, but with differences in the individual phenolic response [208]. Such a specificity of response by different molecules was observed also in chili pepper (*Capsicum annuum*, cv. Coronel) plants exposed to 4-h-daily supplemental UV-B radiation (80 mW m^−2^, UV-B broadband lamps as lighting source), which underwent an increase in the content of chlorogenic acid, luteolin 8-C-hexoside, and apigenin 8-C-hexoside both after 7 and 14 days of treatment, while apigenin 6-C-pentoside-8-C-hexoside accumulated only after 14 days of UV-B exposure [210].

Other examples of the complexity of the phenolic response to UV-B radiation are present in literature. For example, the positive effect of UV-B in wheat seedlings depended on the moment and the duration of application of UV-B irradiation (0.1 W m^−2^, UV-B lamps as lighting source) after the seeds germination [206]. A heterogeneity of response in relation to the UV-B dose applied (0.205 W m^−2^, from 0.5 h to 3.5 h, UV-B lamps as lighting source) was observed in mung bean (*Vigna radiata*) sprouts. Indeed, the shortest irradiation periods induced a decrease in phenolic content, which contrarily increased over the control level following 2.5-h exposure. However, flavonoid content increased regardless the UV-B dose [207].

**Table 5:** Biochemical responses of crops and plants of food interest to UV-B wavelengths considered in this review. Tot, total phenolics; Flav, flavonoids; Ant, anthocyanins; AC, antioxidant capacity; T, terpenoids; AA, ascorbic acid; TP, tocopherols; GSL, glucosinolates. For each plant species and cultivar, and for each secondary metabolite or metabolic class considered, the symbols “↓”, “↑” and “=” mean a decrease, increase or no variations, respectively, compared to the control plants of each study.

Species	Cultivar	Phenolics			AC	T	AA	TP	GSL	Ref.
		Tot	Flav	Ant						
Basil (*Ocimum basilicum* L.)	Genovese	↑/=				↑/=				[200,201,202,203]
	Cinnamon					↑/↓	↑/=/↓			[211]
Rice (*Oryza sativa* L.)	Kanchana	↑				↑				[204]
	Mattatriveni					↓/=				
	Harsha					↑/=				
Broccoli (*Brassica oleracea* L. var. *italica*)	Waltham 29	=				↑/=			↑	[185,205,212]
Broccoli (*Brassica oleracea* var. *gemmifera* DC)									=	[187]
Wheat (*Triticum aestivum* L.)	Sumai188	↑								[206]
Mung bean (*Vigna radiata*)		↑/↓	↑				↑			[207]
Peppermint (*Mentha piperita* L.)	Rubescens	↑				↑/↓				[208]
Lettuce (*Lactuca sativa.* L.)	Red leaf cvs.	↑	↑	↑		↓				[191,209]
	Green leaf cvs.	↑	↑			↑				
Peach fruit (*Prunus persica* L.)	Suncrest	↑/=/↓	=	↓/↑			=/↓			[213,214]
	Big Top	↑/=/↓	↓/↑	↑						
	Babygold 7	↓	↓/↑	=						
	Fairtime	↓/↑	↓/↑	↓/↑		↓				[215,216]
	Yulu			↑						[199]
	Hujingmilu			↑						
Tomato fruit (*Solanum lycopersicum* L.)	Money Maker					↑	↑			[217,218]
	Zhenfen 202						↓			[219]
Bell pepper fruit (*Capsicum annum* L.)	Angus					↑				[220]
Green lime fruit (*Citrus latifolia* Tan.)						↑				[221]
Spinach (*Spinacia oleracea* L.)	Meridian							↑		[222]
Maize (*Zea mays* L.)								↓		[223]
Cucumber (*Cucumis sativus* L.)	Long green							↓		[224]
Apple fruit (*Malus domestica* Borkh.)	Aroma						↑			[225]

One of the most evident effects of UV-B on phenolics compounds is the positive influence played on anthocyanins biosynthesis, which leads to enhanced pigmentation of both leafy vegetables and fruits. A bright and intense colour is perceived by consumers as a marker of quality and freshness of the products, and therefore, it orientates food preference and acceptability. Zhao et al. [199] reported a successful increase in cyanidin-3-glucoside levels, accompanied by a reddenish, in the peel of peach fruits subjected to UVB (280–315 nm, 0.58 W m^−2^) irradiation for 2 days. This response was more evident in cv. Yulu than in cv. Hujingmilu, the latter also being responsive to UV-A radiation. Anthocyanin accumulation was paralleled by a coordinated upregulation of genes involved in anthocyanin biosynthesis.

Another study carried out on post-harvest peach (cvs. Suncrest and Babygold 7) and nectarine (cv. Big Top) fruits treated with 1.69 W m^−2^ UV-B (lamp tubes) for 12, 24, or 36 h reported a genotype-dependent response to irradiation. Dose- and structure-dependent effects were also observed. Flavonols and hydroxycinnamic acids of Suncrest and Big Top behaved similarly, decreasing or being unaffected following the lowest dose but accumulating at higher concentration after 24- or 36-h irradiation, respectively. Anthocyanins were generally positively influenced by the treatment irrespective of the UV-B dose, except in Suncrest after 12-h treatment. A quite different response was evident in Babygold 7, where phenolics were generally negatively affected by the treatment [213].

The effectiveness of UV-B radiation in promoting phenolics accumulation in the peel of peach (cv. Fairtime) fruits was confirmed by Santin et al. [216] by exposing the fruits to 2.3134 W m^−2^ UV-B for 10 or 60 min. The authors observed that most metabolites down-accumulated 24 h after the end of the treatment, attributing this effect to their consumption during detoxification of UVB-induced ROS. However, afterwards, an overall increase occurred, particularly evident for anthocyanins, flavones, and dihydroflavonols. Such an increase was attributed to the increased transcription of gene involved in UV-B signalling and in phenylpropanoid biosynthesis (both structural and regulatory genes) occurring 6 h after the treatment [215]. Interestingly, despite UV-B radiation being unable to penetrate below the peach peel [226], the content of pulp phenolics also underwent a general increase. Such an effect was particularly intense for flavanols, flavonols, and flavones, and, differently from what was observed in the peel, it occurred 24 h after the end of the treatment. As in the peel, however, flavonoid biosynthetic and regulatory genes were upregulated by UV-B after 6 h from the irradiation [227]. This finding is particularly interesting because it demonstrates the capability of UV-B treatments in improving the nutraceutical properties of peach pulp—that is the only part usually consumed due to the custom of peeling the fruit.

### 9.2. Terpenoids and Chlorophylls

The studies carried out on the impact of UV-B radiation on terpenoids are less numerous than those dealing with phenolics. However, as described for the other wavelengths in the previous chapters, also UV-B can influence this metabolic class. In broccoli sprouts, exposure to UV-B radiation (7.16 W m^−2^) for 120 min induced a significant accumulation of lutein and neoxanthin [205]. In the same plant species, however, β-carotene was unchanged 24 h after the end of the UV-B irradiation (0.042 Wh m^–2^, 4 h) [212], probably due to the lower dose applied. UV-B supplementation (9 days) during the growth of lettuce plants already receiving UV-A radiation resulted in a different effect in green- or red-leafy cultivars. Specifically, the response was generally positive in the eight green leaf cultivars, except for neoxanthin content of one only cultivar. Conversely, all eight red leaf cultivars underwent a significant depletion of lutein, neoxanthin, and β-carotene [191].

The capacity of UV-B to affect carotenoid content was also demonstrated in fruits. Tomato fruits (cv Money Maker) harvested at a mature green or turning stage, and allowed to ripen under 1 h daily UV-B radiation (1.69 W m^−2^), exhibited a higher carotenoid content in the peel, which was independent from the harvesting stage (and therefore from the duration of the treatment) for β-carotene and lycopene, but not for lutein. Interestingly, lycopene increased also in the fruit flesh. However, the positive effect observed in the peel of cv. Money Maker was not detected in the photomorphogenic *hp-1* mutant, characterised by a constitutively high pigmentation [217]. Differently, a metabolomic analysis of the peel of peach (cv. Fairtime) fruits treated in post-harvest with 2.3134 W m^−2^ UV-B for 10 or 60 min revealed a decreased concentration of several carotenoids in treated fruits 36 h after the end of the irradiation. The authors hypothesised a consumption of these molecule to counteract the UV-B-induced ROS formation, which could be followed by a later accumulation due to stimulation of biosynthetic genes, similarly to what detected for phenolics in the same fruits [216]. Consistent with this hypothesis, the same treatment (60 min irradiation), induced an increase of carotenoid concentration in the peach pulp already after 24 h of recovery, probably because the UV-B radiation, not passing through the outer skin, did not caused ROS production in the flesh beneath [226]. A positive influence of UV-B radiation on carotenoid accumulation was also reported in bell pepper (*Capsicum annum* L., cv. Angus) fruits, exposed to UV-B radiation (8.94 W m^−2^, 19 min and 30 sec) 6 days after the harvest, and then kept under different light conditions for up to 4 days to simulate a retail sale period. The carotenoid profile highlighted that such a positive effect was particularly evident when UV-B treatment was followed by storage under fluorescent/blue–red LED day/night photoperiod [220].

A deep understanding on the UV-B-induced modulation of terpenoids represents a crucial aspect especially from an applicative point of view, since the terpenoid class include most volatiles that contribute to the aroma of plants and fruits of food interest. Hence, the consequent modification of organoleptic properties might alter the consumers’ attitude towards the UV-B-irradiated plant products. A 1-h daily UV-B irradiation (UV-B broadband lamps as lighting source) on basil plants up to 15 days determined a significant increase in essential oil content (whose terpenoids were mainly represented by monoterpenes linalool and 1,8-cineole) both in mature and developing leaves, increasing the aroma of basil plants [202]. Another study on basil, treated with UV-B radiation (2.23 W m^−2^, 3 h per day, UV-B broadband lamps as lighting source) for two weeks, found a significant increase in linalool and 1,8-cineole, although most of the volatile molecules did not vary after the treatment [203]. Similarly, a positive effect of a two-weeks UV-B treatment (UV-B broadband lamps as UV-B source) on basil plants was found considering the predominant essential oil components (e.g., linalool, 1,8-cineole, and trans- β-ocimene) [228]. UV-B radiation was also able to modulate terpenoid content in peppermint plants grown either in open field or in growth chambers. A 1-h UV-B irradiation (310 nm, 7.1 kJ m^−2^ d^−1^ UV_BE_, UV-B broadband lamps as UV-B source) performed some days before full bloom resulted in modulating the profile of essential oil and particularly the volatile compounds [208], with differences in the trend of response by specific volatiles. Considering the great importance of peppermint essential oil in many applicative aspects (e.g., cosmetic, flavouring, and medicinal), the possibility to alter both quality and quantity of singular constituents by inserting the UV-B radiation during plant growth deserves deeper studies.

Increased chlorophyll *a* content was observed in broccoli sprouts exposed to UV-B (7.16 W m^−2^) for 120 min [205] as well as in green-leaf lettuce cultivars grown in the presence of supplemental UV-B radiation [191]. However, also chlorophyll displayed a genotype-dependent response to this wavelength, as demonstrated by the decrease in its content in the red-leaf cultivars of lettuce [191]. Different results were obtained in basil as well. Indeed, Nascimento et al. [201] did not observe any variation in both chlorophyll *a* and *b* content of cv. Genovese cultivated under white light supplemented with 0.5 W m^−2^ UV-B (8 h per day, 4, days, UV lamps). Conversely, cv. Cinnamon exposed to a similar irradiance (0.571 W m^−2^, 1 or 2 h per day, 7 days) accumulated more chlorophylls as compared to control when treated at a juvenile stage (3–4 leaf pair growth), but underwent a chlorophyll depletion when irradiated at the flowering stage [211]. Therefore, genotype differences, but also the plant developmental stage, as well as UV-B dose, may influence the plant capacity to respond to irradiation.

In fruits, UV-B radiation (7.33 W m^−2^) was proven to delay chlorophyll degradation in mature green lime (*Citrus latifolia* Tan.) fruits when administered for 20 min. The content of both chlorophyll *a* and *b* of treated fruits remained stable for up to 30 days of storage, differently from control fruits where both pigments started to decrease after 15 days. Differently, a 30-min exposure induced fruit yellowing [221]. Therefore, if adequately calibrated, UV-B treatments can be a valid method to prevent the loss of marketable quality during storage.

### 9.3. Other Secondary Metabolites

The potential of UV-B radiation to increase the content of health-promoting compounds other than phenolics and terpenoids was also studied, though reports present in literature are still scarce. In particular, to the best of our knowledge, UV-B influence on tocopherols content of fruits and vegetables was not investigated, though an increased accumulation of α-tocopherol was detected in thylakoid membranes of spinach (*Spinacia oleracea* L. ‘Meridian’) plants irradiated with supplemental UV-B radiation (9 h daily for 12 d, 13.5 kJ m^–2^ d^–1^ of UV-B_BE_) [222], while in maize (*Zea mays* L.) seedlings, as well as in cucumber (*Cucumis sativus* L. cv. Long green) cotyledons, UV-B exposure (8.35 kJ m^−2^ UV-B_BE_ per day for 9 days, or 0.2 W m^−2^ for 1 h per day for 3 days, respectively) was found to induce a significant decrease in α-tocopherol content [223,224].

UV-B radiation was found to be effective also in modulating ascorbic acid concentration. Mung bean seedlings exposed to eight UV-B exposure times, from 0 to 3.5 h (0.205 W m^−2^, UV-B lamp as UV-B source) showed an enhanced content of ascorbic acid in the 0.5-h, 2-h and 2.5-h treatments [207]. In basil leaf, the UV-B effect depended on plant age and radiation dose: in young plants, the lowest dose, but not the highest one, induced an accumulation of ascorbate, while, at the flowering stage, ascorbic acid decreased or increased following the lowest or the highest UV-B dose, respectively [211].

The influence of UV-B on ascorbate content was observed also in peach [214] and tomato [218] fruits. In both cases, such effect was evident not only in the peel, but also in the flesh beneath, although not directly reached by UV-B radiation. In particular, in peach (cv. Suncrest) post-harvest irradiation (1.69 W m^−2^ UV-B) for 12 h led to a depletion ascorbic acid in both skin and flesh, but it induced an accumulation in the skin when applied for 36 h. However, regardless of the trend of variation, no change in ascorbate redox state occurred following UV-B irradiation [214]. An increased content of ascorbic acid was previously detected also in shade-grown apples (cv. Aroma) peel, but not in the sun-exposed ones, following 10 days of post-harvest treatment with visible light + 0.17 W m^−2^ UV-B for 12 h per day in comparison to dark-stored fruits [225].

A different response attributable to genotype differences was observed in tomato fruits, where UV-B post-harvest treatment (1.69 W m^−2^, 1 h per day until full ripening) induced an increased accumulation in both skin and flesh of Money Maker fruits, irrespective of the harvesting stage (mature green on turning), while in the *hp-1* mutant an unchanged content was observed in the peel and a slight decrease occurred in the flesh of fruits irradiated at the mature green stage [218]. The genotype-dependence of ascorbate reaction to UV-B radiation is confirmed by the findings of Liu et al. [219] who observed a lower content of this antioxidant compound in UVB-treated tomato (cv. Zhenfen 202) fruits harvested at the MG stage and subjected to irradiation with different UV-B doses. In addition to the genotype effect, the mode of irradiation undoubtedly contributes to different outcomes, as exemplified by the comparison of these last two articles, with the former applying a long-term exposure to low-intensity UV-B, and the latter irradiating the fruits with an acute application at the beginning of the storage period.

Studies were published on the positive effect played by UV-B radiation on glucosinolates. Exposing broccoli sprouts (cv. Waltham 29) to UV-B radiation (280–320 nm, 7.16 W m^−2^, UV-B broadband as lighting source) for 120 min led to a significant increase in total glucosinolate concentration, and in particular glucoiberin, glucoraphanin, 4-hydroxy-glucobrassicin, glucoerucin, glucobrassicin, and 4-methoxy-glucobrassicin [205]. Lower irradiations (2.28 and 3.34 W m^−2^ for 120 min) were effective as well in increasing both indole and aliphatic glucosinolates of the same cv. of broccoli sprouts, particularly 2 and 24 h after the end of the irradiation, for the lowest and the highest dose, respectively [185]. Similarly, Mewis et al. [212], always in broccoli sprouts, detected an increase in the content of these metabolites even following a single and low UV-B dose (0.042 Wh m^–2^, 120 min), and did not find a greater accumulation of the aliphatic glucosinolates by applying higher doses or by replicating the single-dose exposure. Moreover, broccoli florets accumulated more glucosinolates in response to UV-B irradiation (20.4 W m^−2^, at the dose of 1.5 and 7.2 kJ m^−2^), particularly those belonging to the indole class, such as glucobrassicin. The increased glucosinolate content was accompanied by overexpression of some biosynthetic genes [229].

All of these examples highlight the general positive effect played by UV-B radiation on glucosinolates, with some differences attributable to the irradiation dose. Acharya et al. [187] reported instead on the absence of any variation in both indole and aliphatic classes following irradiation of broccoli sprouts with UV-B LEDs (300 nm, 0.03 kJ m^−2^ d^−1^, corresponding to 0.35 mW m^−2^) and the authors attributed the results to the low efficiency of UV-B LEDs, in respect to the UV-B emitting tubes used in the other experiments.

## 10. Conclusions

The light-induced modulation of plant secondary metabolism has gained great attention within the last decades, particularly in regard to enhancing the nutraceutical value of fruits and vegetables. In light of ever-increasing consumer demands (i.e., of health-promoting plant-based foods), application of light treatments is considered a sustainable and eco-friendly way to achieve this goal, both pre- and post-harvest. Biosynthesis of any molecular class addressed in this review was found regulated by light. Though some responses of a certain class of bioactive compounds to a specific wavelength were recorded more frequently than others (i.e., accumulation vs. depletion), one common feature of the light influence on secondary metabolites, irrespective of the spectral radiations considered, is the species- or cultivar-dependent specificity of response. The genetic background witnesses the evolutionary pressure experienced by a plant. Specifically, the predominant light environment during evolution had led to the selection of different degrees of sensitivity to light intensity and quality, and resulted in different abilities to adapt to specific habitats. Geographical origin of the plant is therefore important in this context, though, in the case of domesticated species/cultivars, the breeding history (use of landraces or modern breeding programs) is of pivotal importance as well. Moreover, the radiation intensity and duration of exposure, as well as the chemical structure of compounds belonging to the same molecular class, were often important determinants of the output. In greenhouses, light has long been controlled and designed on specific crops to improve and standardize production, but the need to adopt a light environment that also improves the nutraceutical quality of products is now becoming even more important. This aspect is also critical in post-harvest when the products undergo an inevitable qualitative impoverishment during storage. The development of high-performing LEDs and the setup of specifically designed experiments will make it possible to furnish lighting recipes and irradiation protocols to optimize the production of plant foods with high added value.

## Data Availability

No new data were created or analysed in this study. Data sharing is not applicable to this article.
